# Vast diversity of prokaryotic virus genomes encoding double jelly-roll major capsid proteins uncovered by genomic and metagenomic sequence analysis

**DOI:** 10.1186/s12985-018-0974-y

**Published:** 2018-04-10

**Authors:** Natalya Yutin, Disa Bäckström, Thijs J. G. Ettema, Mart Krupovic, Eugene V. Koonin

**Affiliations:** 1 National Center for Biotechnology Information, National Library of Medicine. National Institutes of Health, Bethesda, MD 20894 USA; 2 Department of Cell and Molecular Biology, Science for Life Laboratory, Uppsala University, Box 596, -75123 Uppsala, SE Sweden; 3 Unité Biologie Moléculaire du Gène chez les Extrêmophiles, Department of Microbiology, Institut Pasteur, Paris, France

## Abstract

**Background:**

Analysis of metagenomic sequences has become the principal approach for the study of the diversity of viruses. Many recent, extensive metagenomic studies on several classes of viruses have dramatically expanded the visible part of the virosphere, showing that previously undetected viruses, or those that have been considered rare, actually are important components of the global virome.

**Results:**

We investigated the provenance of viruses related to tail-less bacteriophages of the family *Tectiviridae* by searching genomic and metagenomics sequence databases for distant homologs of the tectivirus-like Double Jelly-Roll major capsid proteins (DJR MCP). These searches resulted in the identification of numerous genomes of virus-like elements that are similar in size to tectiviruses (10–15 kilobases) and have diverse gene compositions. By comparison of the gene repertoires, the DJR MCP-encoding genomes were classified into 6 distinct groups that can be predicted to differ in reproduction strategies and host ranges. Only the DJR MCP gene that is present by design is shared by all these genomes, and most also encode a predicted DNA-packaging ATPase; the rest of the genes are present only in subgroups of this unexpectedly diverse collection of DJR MCP-encoding genomes. Only a minority encode a DNA polymerase which is a hallmark of the family *Tectiviridae* and the putative family "Autolykiviridae". Notably, one of the identified putative DJR MCP viruses encodes a homolog of Cas1 endonuclease, the integrase involved in CRISPR-Cas adaptation and integration of transposon-like elements called casposons. This is the first detected occurrence of Cas1 in a virus. Many of the identified elements are individual contigs flanked by inverted or direct repeats and appear to represent complete, extrachromosomal viral genomes, whereas others are flanked by bacterial genes and thus can be considered as proviruses. These contigs come from metagenomes of widely different environments, some dominated by archaea and others by bacteria, suggesting that collectively, the DJR MCP-encoding elements have a broad host range among prokaryotes.

**Conclusions:**

The findings reported here greatly expand the known host range of (putative) viruses of bacteria and archaea that encode a DJR MCP. They also demonstrate the extreme diversity of genome architectures in these viruses that encode no universal proteins other than the capsid protein that was used as the marker for their identification. From a supposedly minor group of bacterial and archaeal viruses, these viruses are emerging as a substantial component of the prokaryotic virome.

**Electronic supplementary material:**

The online version of this article (10.1186/s12985-018-0974-y) contains supplementary material, which is available to authorized users.

## Background

The last several years have witnessed major advances in our understanding of the diversity of the global virome (the entirety of viruses in the biosphere) and the evolutionary relationships among viruses and virus-like mobile genetic elements (MGE). As of late, the principal methodology for discovery of new viruses has dramatically changed: instead of the traditional virus isolation and cultivation, the great majority of new viruses are now discovered through metagenomic sequence analysis [1–7]. Some of these studies drastically change the existing knowledge on the virome compositions in various habitats. One of the most striking cases in point is the discovery of crAssphage, by far the most abundant virus in the human intestine and generally, in the human-associated virome that remained unknown until the advent of metagenomics [8–10]. At the time of the initial discovery, most of the crAssphage genes remained uncharacterized, and no related viruses have been identified, so that, despite its ubiquity and high abundance in humans, this virus remained completely enigmatic and recalcitrant to further investigation [9]. However, a follow up study taking advantage of expanded databases and sensitive homology detection methods has led to the identification of an expansive family of bacteriophages all of which are predicted to infect bacteria of the phylum Bacteroidetes [11]. The main structural and replication genes of these viruses have been identified, making them amenable to experimental characterization.

Metagenomic sequence mining has led to the discovery of previously unrecognized groups of viruses that apparently infect uncultivated bacteria and archaea, and are likely to be important ecological players. An example is a novel family of viruses associated with uncultivated Group II marine archaea, where both the hosts and the viruses appear to be among the most common members of the ocean that would remain obscure without the metagenomic effort [12, 13]. Other metagenomic studies have drastically changed the status of certain groups of viruses that previously have been considered minor components of the virosphere. In particular, metagenomic analyses have revealed enormous, unsuspected diversity and abundance of single-stranded (ss) DNA viruses [14–18]. These are only a few of the metagenomic discoveries which collectively indicate that traditional methods for virus isolation have only scratched the surface of the virosphere, whereas the actual diversity and structure of the global virome can be characterized only by comprehensive metagenomic analyses. In recognition of this sea change in virus research, the International Committee for Taxonomy of Viruses (ICTV) is now accepting proposals for new virus species and higher taxa on the basis of metagenomic sequences alone [7].

Parallel to the advances in metagenomics, and in large part, fueled by metagenomic discoveries, there has been considerable progress in the reconstruction of virus evolution. The major emerging trend is the ultimate modularity of virus evolution whereby evolutionarily coherent structural and replication modules combine promiscuously with one another and with a variety of additional genes [19–21]. One of the most notable cases in point are the ssDNA viruses that appear to have evolved on multiple occasions via independent recombination events between a capsid protein gene derived from RNA viruses and a plasmid replication module [20, 22, 23]. A complete reconstruction of virus evolution is feasible only through phylogenomic analysis of both the structural and the replication modules, along with the recombination events [24]. In practice, the structural module is often the best marker of virus evolution because the structural genes seem to be exchanged or eliminated less often than replication genes, and hence provide for unification of more diverse groups of viruses [25, 26].

The great majority of the double-stranded (ds) DNA viruses with moderate-sized and large genomes can be partitioned into two vast supergroups with distinct, unrelated structural modules [21]. The robustness of the two groups has been validated quantitively by analysis of bipartite, gene-genome networks [27]. The first supergroup includes most of the known head-tail bacteriophages (order *Caudovirales*), a variety of phage-like viruses infecting mesophilic archaea, and the animal viruses of the order *Herpesvirales*. All these viruses possess icosahedral particles formed by the so called HK97 fold (named after the eponymous bacteriophage) capsid protein and a two-subunit terminase that mediates ATP-dependent DNA packaging into the capsid. The second supergroup consists of two families of bacteriophages (*Tectiviridae* and *Corticoviridae*) [28], archaeal viruses of the family *Turriviridae* [29] and many diverse groups of eukaryotic viruses including giant eukaryotic viruses of the putative order “Megavirales” [30]. All these viruses also possess icosahedral capsids that, however, are built of the double jelly-roll major capsid protein (DJR MCP [31, 32]) that is unrelated to the HK97 capsid protein, typically, accompanied by a single jelly roll minor capsid protein. Furthermore, these viruses employ a distinct ATPase that belongs to the FtsK-HerA superfamily of P-loop NTPases [33] and is unrelated to the terminase, for DNA packaging.

The two major supergroups of dsDNA viruses strongly differ with respect to the representation of viruses infecting prokaryotes and eukaryotes. The HK97 supergroup consists primarily of prokaryotic viruses, the tailed phages that represent a substantial majority among all known viruses. By contrast, viruses of eukaryotes are represented by a single, even if expansive, order *Herpesvirales*, with representatives so far detected only in animals. In contrast, viruses of the DJR MCP supergroup attained remarkable diversity in eukaryotes but are only sparsely represented among the known viruses of prokaryotes. We sought to explore the actual expanse of the DJR MCP group among prokaryotes by searching genomic and metagenomic databases for homologs of the tectivirus-like MCP using sensitive sequence analysis methods. In genomes and metagenomes from various environments, we discovered numerous, highly diverse DJR MCP-encoding sequences in variable genomic contexts. Analysis of these sequences revealed several groups of previously unknown viruses and proviruses that show extreme plasticity of gene repertoires and genome organizations.

## Methods

### Genomic and metagenomic database screening

The sequences of the DJR MCPs of previously identified prokaryotic viruses and proviruses, namely:

-Enterobacteria phage PRD1 (NP_040692).
-Pseudoalteromonas phage PM2 (NP_049903).
-Bacillus phage Bam35c (NP_943764),
-Sulfolobus turreted icosahedral virus (YP_025022).
-Methanococcus voltae provirus (ADI36123).
-Thermococcus kodakarensis provirus (BAD85542).
-Flavobacterium phage FLiP (ASQ41214).

were used as queries for PSI-BLAST [34] searches against the GenBank non-redundant protein sequence database (nr) and for TBLASTN [34] searches against the metagenomic database (wgs) at the NCBI [35]. The sequences of putative new MCPs retrieved in these searches were aligned with the original query sequences using MUSCLE [36]. The newly detected sequences were validated as bona fide MCP homologs by inspection of the conserved structural elements and secondary structure prediction, and the multiple sequence alignments were used as queries for additional database searches using PSI-BLAST and HHpred [37]; the search procedure was iterated until convergence. In addition to the contigs from public databases, new contigs from Loki’s Castle (hydrothermal vent field on the Arctic Mid-Ocean Ridge from which diverse archaea have been isolated including the closest known archaeal relative of eukaryotes) were analyzed (denoted Loki_contigs in the figures). In the process of reconstructing the genomic bins of the Asgard archaea superphylum, 232.3 Gbp of sequence data from a deep sea sediment sample near Loki’s Castle was assembled using different assembly programs and parameters [38]. The DJR MCP proteins identified as described above were used to create a HMM profile with HMMER 3 [39], and these profiles were used to search Loki’s Castle assemblies. The assembly that was chosen to extract the sequences was assembled using Ray Meta [40], with a k-mer size of 45 which was identified as optimal for yielding the longest contigs matching the MCP query [38].

### Sequence analysis of MCP-encoding contigs

Protein coding sequences in contigs obtained as described above were predicted using GeneMark hmm prokaryotic and translated (version 3.25) [41]., The resulting proteins sequences were used as queries to search the nr database using PSI-BLAST, the Conserved Domain Database (CDD) using RPS-BLAST [42] and the PDB, CDD and Pfam databases using HHPred [37]. For poorly characterized proteins, multiple alignments were constructed using MUSCLE, profiles were constructed from the resulted multiple alignments. Additional searches of the same database were performed using these profiles as queries for PSI-BLAST and HHpred, in an attempt to identify homologs with low sequence similarity to the query proteins. This procedure was terminated when such homologs were confidently identified, but in cases where none were found, was iterated until convergence. The MCP sequences were clustered by similarity (BLOSUM62 matrix, E = 10^− 03^) using the CLANS program which generates a network representation of pairwise sequence similarities between proteins using a version of the Fruchterman-Reingold graph layout algorithm [43].

### Phylogenetic analysis

Protein sequences were aligned using MUSCLE, and poorly aligned (low information content) positions were removed [44]. Phylogenetic trees were constructed using the FastTree program, with default parameters [45].

## Results

### Search of genomic and metagenomic sequence databases for prokaryotic viruses encoding DJR MCP

**Fig. 1:**
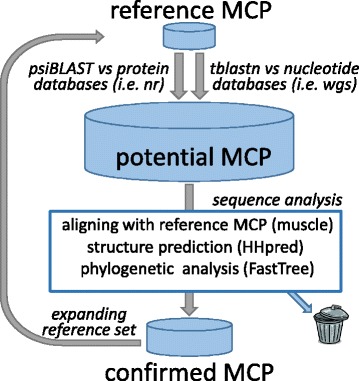
The iterative procedure for detecting DJR MCP-encoding contigs in genomic and metagenomic databases.

The sequences of the DJR MCP of previously identified prokaryotic viruses and proviruses were used as queries for PSI-BLAST searches against the GenBank non-redundant protein sequence database (nr) and TBLASTN searches against the metagenomic database (wgs). The sequences of putative new MCP retrieved in these searches were aligned with the original query sequence. After validation of the new sequences by inspection of the conserved structural elements and secondary structure prediction, the multiple alignments of several groups of DJR MCP were used as queries for additional database searches using PSI-BLAST and HHpred, and this procedure was iterated until convergence. Clearly, the possibility that even more divergent MCP variants are represented in the current databases but were missed in our analysis cannot be ruled out and, actually, appears likely. The identified MCP-encoding contigs were extracted from the database, the protein-coding genes were predicted (or extracted from GenBank whenever available), and the encoded protein sequences were analyzed using PSI-BLAST, HHpred and, when appropriate, phylogenetic trees were constructed. The workflow for the discovery of DJR MCP encoding genomes is shown in Fig. 1 (see Methods for further details).

**Fig. 2:**
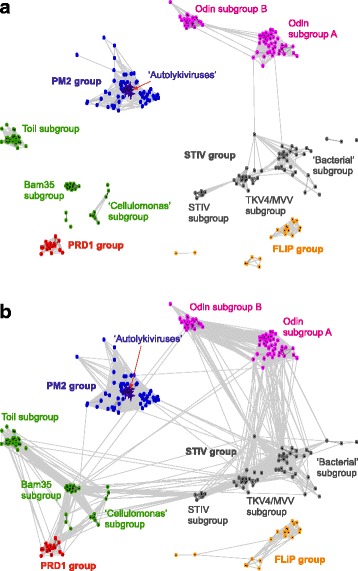
Sequence similarity networks of prokaryotic virus DJR MCP. Protein sequences were clustered by the pairwise sequence similarity using CLANS. Different groups of DJR MCP are shown as clouds of differentially colored circles, with the corresponding subgroups labeled as indicated in the text.**a**. Stringent threshold (CLANS *P*-value ≤1e-10). Members of the putative family “Autolykiviridae” [77] that represent a tight cluster within the PM2 group are shown by purple stars and indicated by an arrow. **b**. Liberal threshold (CLANS P-value <1e-03)

Altogether, we identified 524 contigs encoding DJR MCPs that were partitioned into 6 groups based on the results of the comparative analysis of the MCP sequences and gene content. Sequence similarity clustering analysis using CLANS (see Methods) with a stringent cut-off (*P* < 10^− 10^) yielded 7 tightly connected clusters (Fig. 2a) which we denoted, after the representative experimentally characterized viruses (with the exception of the Odin group which included no such members): 1) Odin group, with 2 distinct subgroups, 2) PM2 group, 3) STIV group, with 3 subgroups, 4) Bam35 group, 5) Toil group, 6) PRD1 group, 7) FLiP group. Additionally, there were several isolated small clusters. Subsequent more detailed analysis using profile-based methods (PSI-BLAST and HHpred along with examination of the genome organizations led us to merge the Bam35 and Toil (sub)groups that consist of biologically similar tectiviruses with similar genome organizations (see below). Additionally, the small clusters were merged into the Bam35-Toil group, STIV group or FLiP group. When a permissive cut-off (*P* < 10^− 3^) was used, the MCPs formed a strongly connected network (Fig. 2b). However, the FLiP-like MCP formed a separate cluster which could not be connected to any other group of DJR MCP at this threshold. The separation of the FLiP group is not unexpected because it includes extremely diverged DJR MCP encoded by ssDNA viruses as opposed to the dsDNA genomes in all other groups (see below). All other MCP groups formed an interconnected network, with some of the groups, such as Bam35-like MCP, connecting to several other groups. The PM2-like viruses were most loosely connected to the rest of DJR MCP (connectivity was lost at *P* ≤ 1e-05), consistent with the results of structural comparisons [46]. The strongly-connected TKV4-MVV-like subgroup of the STIV group included viruses associated with diverse archaeal lineages, including Euryarchaeota, Crenarchaeota and Thaumarchaeota, suggesting an ancient association of these viruses with archaea. Consistently, the bacterial members of the STIV group were more divergent and more loosely connected to the TKV4-MVV-like subgroup. By contrast, bacterial and archaeal sequences were intermixed within the Odin subgroups A and B, suggesting horizontal virus transfer between bacteria and archaea.

Five of the identified groups of (putative) viruses encoding DJR MCP include prototype genomes of previously characterized viruses, whereas the Odin group is new. Many of the contigs were flanked by inverted or direct repeats and appear to represent complete, extrachromosomal viral genomes. Below, we present detailed genomic analysis of each of the 6 groups.

### Odin group

**Fig. 3:**
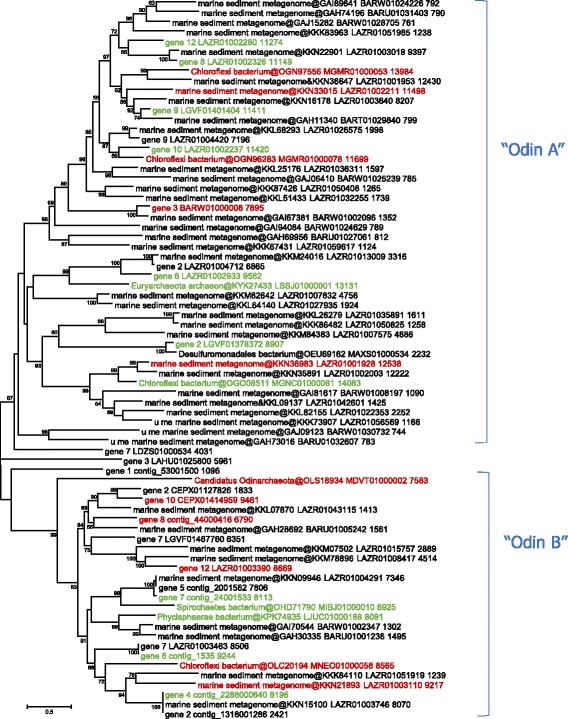
Phylogenetic tree for the Odin group MCP. Genbank protein IDs are shown after ‘@’ (whenever available), followed by GenBank nucleotide ID and contig length (nt). Sequences of the new contigs from Loki’s Castle sediments are denoted ‘Loki_contig_xxx’. The numbers at the internal branches indicate local likelihood-based support (percentage points). The root was arbitrarily enforced between the two major subgroups, denoted A and B. Contigs selected for genome analysis are colored green or red (Additional file 2); contigs selected for Fig. 3 are marked with red

**Fig. 4:**
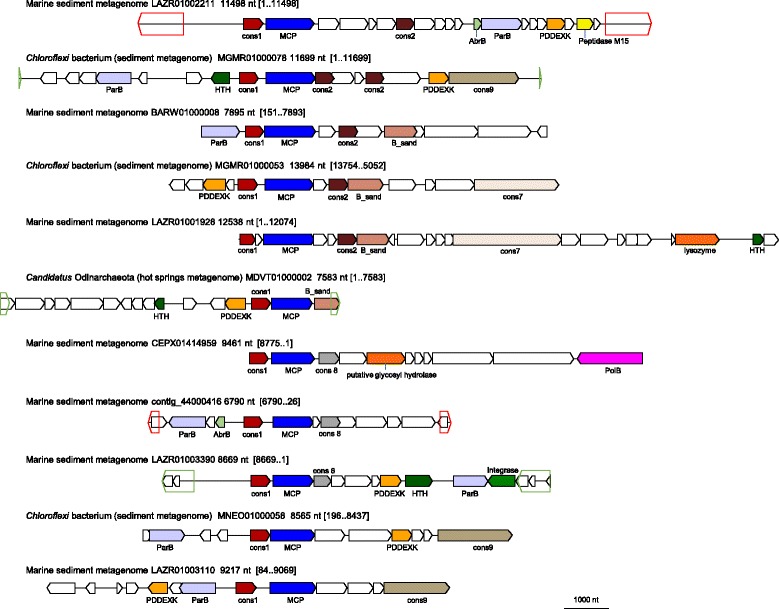
Genome maps for selected members of the Odin group. Empty pentagons and triangles denote direct (green) and inverted (red) terminal repeats. Genes are shown by block arrows drawn to scale. Gene abbreviations: cons1, uncharacterized Odin group protein 1; MCP, major capsid protein; cons2, uncharacterized Odin group protein 2; AbrB, AbrB/MazE/SpoVT family protein; ParB, ParB-like nuclease; PDDEXK, PD-(D/E)XK family nuclease; HTH, helix-turn-helix domain-containing protein; B_sand, beta-sandwich jelly-roll fold protein; cons7, uncharacterized Odina group protein 7; cons8, uncharacterized Odin group protein 8; cons9, uncharacterized Odin group protein 9; PolB, DNA polymerase family B

The identified MCPs of this group show strong structural similarity to the MCPs of crenarchaeal turrivirus STIV and bacterial corticovirus PM2 (see Additional file 1). The group unites contigs from marine sediment metagenomes, some of which are currently assigned to bacteria, and others to archaea. One of these contigs come from the same metagenomes from which the Odinarchaeota, one of the phyla of the Asgard archaea, the apparent closest relatives of eukaryotes, have been isolated, and hence the provisional name we coined for this group [38, 47]. The MCP sequences of the Odin group were re-aligned, and a phylogenetic tree was constructed (Fig. 3). The tree splits into two large clades, each containing intermixed sequences associated with archaea and bacteria. From this tree, representative genomes were chosen based on the contig length and the diversity of the predicted gene repertoire. Several of these contigs contain either inverted or direct long nucleotide repeats suggesting that they represent stand-alone virus genomes of 7–11 kb in size; in contrast, in another contig, the putative viral genes are flanked by typical bacterial genes, indicative of a provirus (Fig. 4; see Additional file 2 for more genome maps).

A striking feature of the Odin group is the absence of a detectable packaging ATPase that otherwise appears to be an integral component of the DJR MCP structural module. It seems highly unlikely that a packaging ATPase was missed in our searches because P-loop ATPases are readily identified through the presence of the Walker A and B motifs, even if more precise classification of these ATPases is challenging in many cases [33, 48, 49]. In all these contigs, the DJR MCP gene is preceded by a conserved gene encoding a small protein without detectable sequence or structural similarity to any known proteins (see Additional file 1). Given the conspicuous absence of the packaging ATPase and the typical juxtaposition of the MCP and ATPase genes in other viral genomes ([30] and see below), it seems likely that this protein is essential for DNA packaging into the Odin group virions. However, inspection of the multiple alignment of this small protein shows no conserved polar residues which, together with the small size of the protein, strongly suggests that it has no enzymatic activity. The only other DJR MCP-containing virus lacking a putative genome packaging ATPase is the recently discovered *Flavobacterium*-infecting bacteriophage FLiP [50] (see below), which has a circular ssDNA rather than a dsDNA genome, as is the case for all other known viruses of this class. Thus, these viruses with small genomes apparently employ a distinct mechanism of DNA encapsidation that does not require a virus-encoded ATPase. In this respect, they resemble ssDNA viruses and polyoma/papillomaviruses that all lack a dedicated packaging ATPase.

Other proteins that are conserved in this group but not in the other groups of DJR MCP viruses described here are nucleases of the ParB and PD-(D/E)XK families and 3 uncharacterized proteins, one of which is predicted to adopt a single jelly-roll fold (Fig. 4 and see Additional file 1) and thus might be a minor capsid protein. Some of the typical viral proteins are only sporadically present in viruses of the Odin group including family B DNA polymerase, glycosyl transferase, lysozyme, M15 family peptidase, predicted transcription regulators containing a helix-turn-helix (HTH) domain, and integrase.

### PM2 group

This is the largest group that includes about 60% of the detected putative genomes encoding DJR MCP (see Additional files 3, 4). Most of the contigs in this group contain the MCP and other virus genes embedded within a typical bacterial genomic context, mostly, characteristic of Proteobacteria, i. e. represent prophages. Indeed, numerous bacterial PM2-like prophages have been described previously [51]. Several contigs are flanked by long repeats and are likely to represent complete viral genomes. The only characterized virus in this group is *Pseudoalteromonas* virus PM2, the sole current member of the family *Corticoviridae* [52].

The MCP tree for the PM2 group consists of two major branches one of which includes prophages, whereas the other one represents virus-like contigs (Additional file 3). Because genomes of PM2-like prophages have been analyzed in detail previously [51], we examined in detail only the genomes from the second branch (Additional file 3).

**Fig. 5:**
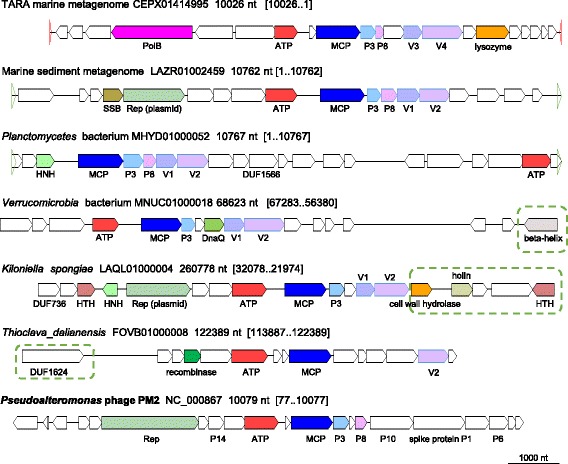
Genome maps for selected members of the PM2 group. Empty triangles denote direct (green) and inverted (red) terminal repeats. Genes are shown by block arrows drawn to scale. Dashed green boxes denote bacterial genes. Gene abbreviations: PolB, DNA polymerase type B; Rep, replication initiation protein; HTH, Helix-turn-helix domain protein; P3 and P8, homologs of PM2 phage structural proteins P3 and P8; V1 and V2, uncharacterized conserved proteins; DnaQ, DnaQ-like exonuclease; HNH, HNH endonuclease; SSB, single-stranded DNA-binding protein; DUF, domain of uncknown function

Only two genes, the MCP and the predicted packaging ATPase that is readily recognizable as a member of the FtsK-like family of P-loop ATPases [33], are conserved among all PM2-like genomes (Fig. 5) [51]. Additionally, some of these putative viral genomes share several conserved genes downstream of the MCP that might encode other virion proteins (Fig. 5). The replication gene block is represented by a rolling-circle replication initiation endonuclease (RCRE) encoded in several contigs and nucleases of different families. Remarkably, one metagenomic contig encodes a DNAP (Fig. 5) that groups with the DNAP of tectivirus PRD1 in the phylogenetic tree, further emphasizing the plasticity within the replication module of the PM2-like viruses (Additional file 5). Furthermore, as in the case of tectiviruses, the latter contig is flanked by terminal inverted repeats, indicative of a complete genome. Collectively, these observations reinforce the evolutionary connection between PM2-like and PRD1-like viruses that has been previously inferred from the conservation of the morphogenetic module alone [46, 53].

### STIV group

This group includes virus-like contigs encoding MCPs with highly diverged sequences such that a single reliable phylogenetic tree could not be constructed. The previously described members of this group (family *Turriviridae*) include two Sulfolobus turreted icosahedral viruses (STIV1 and STIV2), two euryarchaeal proviruses, TKV4 and MVV [54], as well as integrative *Thermococcus nautili* plasmid pTN3 [55] and an extra-chromosomal element of *Pyrobaculum oguniense* [56]. No bacteria-associated members of this group have been reported.

**Fig. 6:**
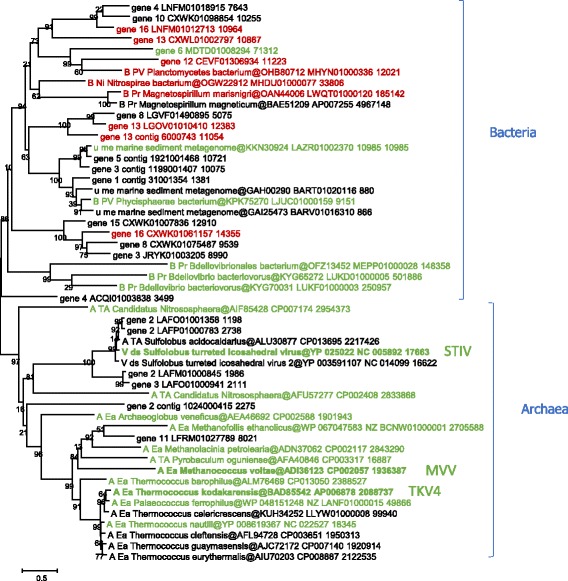
Phylogenetic tree for the STIV group MCP. Genbank protein IDs are shown after ‘@’ (whenever available), followed by GenBank nucleotide ID and contig length (nt). Sequences of the new contigs from Loki’s Castle sediments are denoted ‘Loki_contig_xxx’. The numbers at the internal branches indicate local likelihood-based support (percentage points). The root was enforced between two major subgroups that include, respectively, sequences of bacterial and archaeal origins. Taxa abbreviations: B, Bacteria; A, Archaea; PV, PVC group; Ni, Nitrospirae; Pr, Proteobacteria; Ta, Thaumarchaeota; Ca, Crenarchaeota; Ea, Euryarchaeota. Contigs selected for genome analysis are colored green or red (Additional file 6); contigs selected for Fig. 6 are marked with red. Previously characterized viruses and proviruses are marked with bold font

**Fig. 7:**
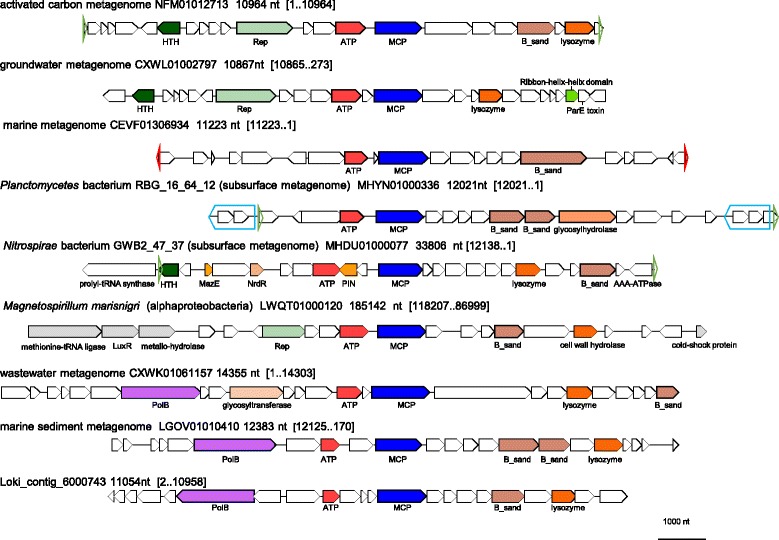
Genome maps for selected members of the STIV group. Empty triangles and pentagons denote direct (green, blue) and inverted (red) repeats. Genes are shown by block arrows drawn to scale. Gene abbreviations: HTH, Helix-turn-helix domain protein; ATP, packaging ATPase; MCP, major capsid protein; B_sand, beta-sandwich jelly-roll fold protein; Rep, replication initiation protein; PolB, DNA polymerase type B; PIN, PIN domain protein; MazE, Antidote-toxin recognition MazE; NrdR, transcriptional regulator NrdR; LuxR, transcriptional regulator LuxR

We identified numerous previously undetected archaeal and bacterial (pro)viruses encoding a DJR MCP related to that of STIV (Figs. 6 and 7, Additional file 6). In particular, additional proviruses similar to TKV4 and MVV were detected in Euryarchaeota and Thaumarchaeota (‘*Candidatus* Nitrososphaera’).

All genomes in this group encode the packaging ATPase highly similar to that of STIV, which has been functionally and structurally characterized [57], although the juxtaposition of the MCP and ATPase genes is not conserved (Fig. 7, Additional file 6). Apart from the MCP and ATPase, no other genes are shared by all members of this group. Many genomes, including STIV, encode integrases of the tyrosine recombinase superfamily that obviously are implicated in provirus integration as well as various nucleases, such as Holliday junction resolvase (TK1364), HNH and PIN-domain endonucleases (Additional file 1). Notably, the integrase of STIV has not been recognized during the original genome annotation [58]. However, HHpred and CD-search analyses indicated that STIV protein A510 (YP_024993) encodes a bona fide member of the tyrosine recombinase superfamily that is homologous to the extensively studied integrases of lambdoid phages (HHpred probability of 99.9 to the integrase of phage lambda). As described previously, TKV4 and MVV possess distinct replication gene modules, with a RCRE gene in MVV and an MCM helicase gene in TKV4 [54]. Among the newly detected members of the STIV group, we identified several with TKV4-like replication genes, several with a distinct variant of MVV-like Rep, and some unique ones including three that encode a DNAP (Fig. 7).

### Bam35-Toil group

**Fig. 8:**
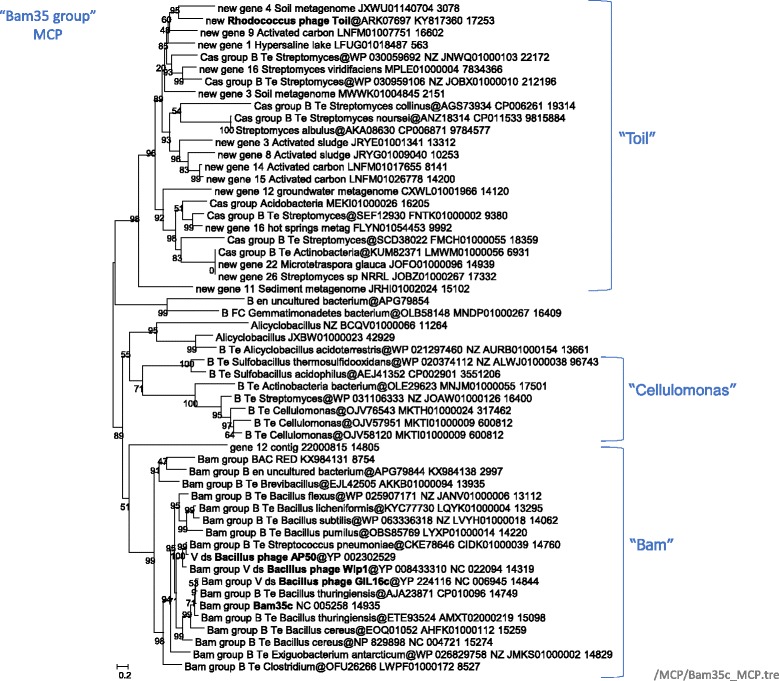
Phylogenetic tree for the Bam35-Toil group MCP. Genbank protein IDs are shown after ‘@’ (whenever available), followed by GenBank nucleotide ID and contig length (nt). Sequences of the new contigs from Loki’s Castle sediments are denoted ‘Loki_contig_xxx’. The numbers at the internal branches indicate local likelihood-based support (percentage points). The root was enforced between the “Toil” subgroup and the rest of the group members. Contigs selected for genome analysis are colored green or red (Additional File 7); contigs selected for the schemes in Fig. 9 are marked with red. Previously characterized phages are marked with bold font

**Fig. 9:**
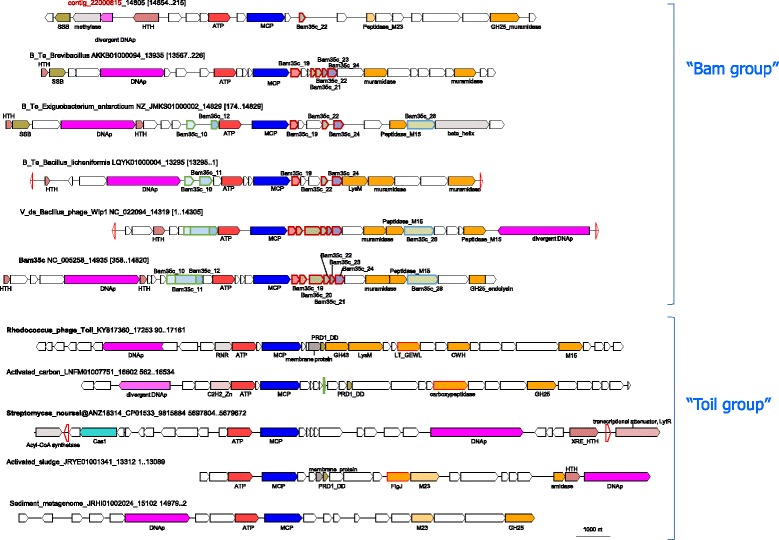
Genome maps for selected members of the Bam35-Toil group. Empty red triangles denoted inverted repeats. Genes are shown by block arrows drawn to scale. Gene abbreviations: HTH, Helix-turn-helix domain protein; ATP, packaging ATPase; MCP, major capsid protein; SSB, single-stranded DNA-binding protein; LysM, LysM domain protein; GH25, Glycosyl hydrolase family 25 (GH25_muramidase); GH43, Glycosyl hydrolase family 43, PRD1_DD, PRD1 phage membrane DNA delivery protein; LT_GEWL, lytic transglycosylase; NlpC/P60 family; RNR, ribonucleotide reductase, monomeric form. Detected homologs of structural proteins of phage Bam35c denoted ‘Bam35c_xx’

This group includes mostly phages and prophages associated with *Terrabacteria* (*Actinobacteria* and *Firmicutes*). Phylogenetic analysis of the MCP and genome comparisons identified two subgroups, which we denote Bam35 and Toil, after the respective bacteriophages (Fig. 8). The Bam35 subgroup includes experimentally characterized tectiviruses Bam35c, Wip1, and AP50 [59–63]. We identified several contigs that likely represent complete genomes of new Bam35c-like phages. In addition to the MCP and ATPase, these putative viral genomes specifically share several genes, including those for minor structural proteins and DNAP, a hallmark of tectiviruses (Fig. 9). Notably, all known Bam35-like phages infect *Bacillus* species. Our results suggest that these viruses are associated with a much broader range of firmicutes, including *Brevibacillus*, *Streptococcus*, *Exiguobacterium*, *Clostridium* species (Fig. 8), consistent with recently published observations [64].

The Toil group is named after the recently isolated *Rhodococcus* phage Toil, a divergent member of the family *Tectiviridae* [65]. These phages and prophages share genes for MCP, ATPase, and lytic enzymes; some also encode DNAPs, including highly divergent, miniaturized variants, and/or predicted transcription regulators (Fig. 9, Additional file 7). Of special interest are two closely related Toil-like proviruses of *Streptomyces noursei* (CP011533) encoding homologs of Cas1, the integrase that is involved in the adaptation (spacer acquisition) by the CRISPR-Cas adaptive immunity systems in archaea and bacteria [66, 67]. Apart from the widespread CRIPSR-Cas systems, Cas1 homologs have been identified in casposons, a distinct family of archaeal and bacterial self-synthesizing transposons in which Cas1 functions as an integrase [68, 69]. The predicted Toil-like prophages in *Streptomyces noursei* and *Streptomyces albulus* have been previously described as Casposons of family 3 because their highly divergent MCP was not recognized at the time [68]. The identification of the MCP makes these elements the first and so far the only viruses encoding Cas1 homologs. In the DNAP phylogeny, the Cas1-encoding elements are embedded among numerous Toil-like viruses and proviruses (Additional file 5), whereas in the Cas1 phylogeny, these elements are nested among capsid-less bacterial casposons [70]. Given the apparent rarity of the Cas1-encoding viruses, it seems likely that these are chimeric elements that evolved via recombination between a casposon and a Toil-like prophage. Presumably, the acquisition of the *cas1-*like genes from casposons enabled the integration into the cellular chromosomes and, accordingly, stable vertical inheritance of the *Streptomyces* prophages in the population. By contrast, the majority of Bam35- and Toil-like prophages lack genes for recombinases and appear to reside in the respective hosts as extrachromosomal linear prophages.

### PRD1 group

**Fig. 10:**
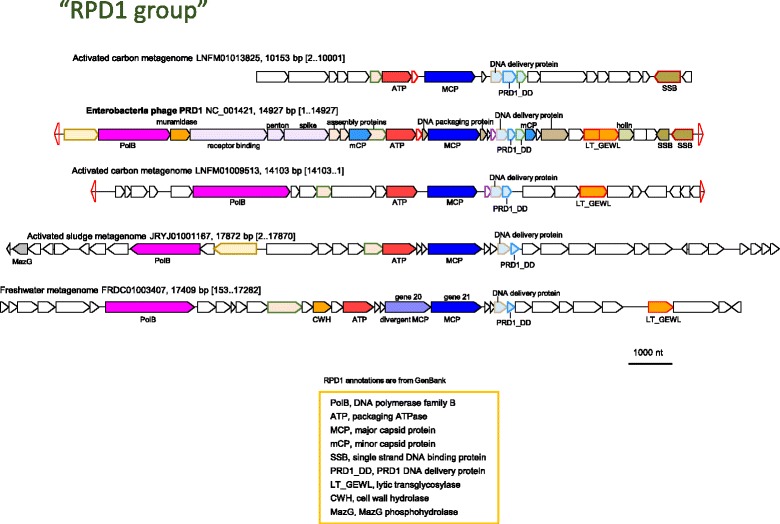
Genome maps for selected members of the PRD1 group. Empty red triangles denote inverted terminal repeats. Genes are shown by block arrows drawn to scale. Gene abbreviations: PolB, DNA polymerase family B; ATP, packaging ATPase; MCP, major capsid protein; mCP, minor capsid protein; SSB, single strand DNA binding protein; PRD1_DD, PRD1 DNA delivery protein; LT_GEWL, lytic transglycosylase; MazG, MazG phosphohydrolase

This group is an extension of the family *Tectiviridae*, named after the most extensively studied and founding member the family, bacteriophage PRD1 [71]. All currently known PRD1-like phages are nearly identical to each other (93–98% nucleotide identity) and infect gram-negative bacteria of the class *Gammaproteobacteria* [28]. We found previously undetected putative PRD1-like virus and provirus genomes in various metagenomic sequences (Fig. 10). Among these contigs, one appears to be a complete virus genome flanked by inverted terminal repeats similarly to the tectiviruses. Interestingly, another contig, FRDC01003407, encodes two copies of the DJR MCP, similar to what has been observed for certain polinton-like viruses [72]. All the detected genomes of the PRD1-like group share a suite of genes, in addition to the MCP and the ATPase, including DNAP, DNA delivery proteins, assembly protein, and lytic transglycosylase. One DNAP is close to that of PRD1 (contig JRYJ01001167), whereas two others are more similar to the DNAP of Toil (contigs FRDC01003407 and LNFM01009513) (Additional file 5). Nevertheless, the newly identified viruses differ from the currently known tectiviruses to a much greater extent than the latter differ from each other (Additional file 8), and might be related to the recently isolated tectivirus GC1 infecting acetic acid bacteria [73].

### FLiP phage-related group of rolling circle-replicating ssDNA viruses

**Fig. 11:**
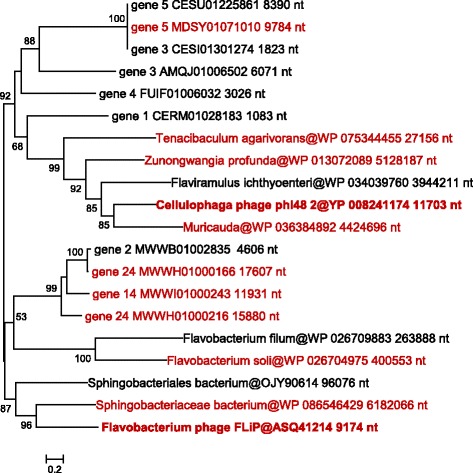
Phylogenetic tree for the FLiP group MCP. Genbank protein IDs are shown after ‘@’ (whenever available), followed by GenBank nucleotide ID and contig length (nt). The numbers at the internal branches indicate local likelihood-based support (percentage points). The root was enforced between two apparent subgroups. The contigs chosen for genome analysis are rendered in red

**Fig. 12:**
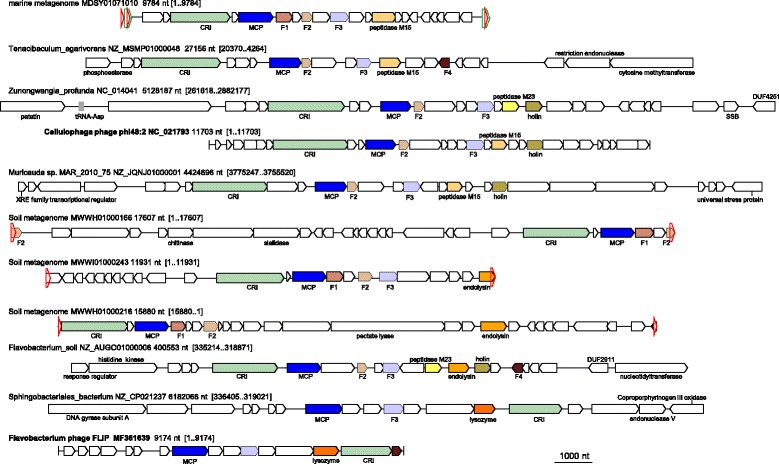
Genome maps for selected members of the FLiP group. Empty triangles denote direct repeats. Genes are shown by block arrows drawn to scale. Gene abbreviations: CRI, Phage replication protein (RCRE); MCP, major capsid protein; F1-F4, uncharacterized proteins; SSB, single-stranded DNA-binding protein

In an effort to obtain a comprehensive census of the DJR MCP viruses in the prokaryotic virome, we also performed database searches using as the query the recently identified DJR MCP encoded by the ssDNA bacteriophage FLiP (*Flavobacterium*-infecting, lipid-containing phage) [50]. The FLiP phage is highly unusual because currently, it is the only identified ssDNA virus encoding a DJR MCP [26]. The fact that the FLiP bacteriophage encodes a DJR MCP has been established by structural analysis of the virus particles, which showed the closest similarity to corticovirus PM2 in both the MCP structure and unique virion geometry (*T* = 21), although no sequence similarity to DJR MCPs of dsDNA viruses could be detected [50]. Our database searches initiated with the FLiP phage MCP sequence also failed to establish any direct connections with previously characterized MCP confirming that these phages encode an extremely diverged version of the DJR fold. However, a group of contigs was detected that encoded homologs of the FLiP MCP along with a distinct variant of the Rep protein homologous to the RCRE of filamentous M13-like inoviruses (Figs. 11, 12). The presence of the M13-like RCRE implies a replication mechanism typical of ssDNA viruses that so far has not been thought to combine with the DJR MCP although phiX174-like RCRE is encoded by some of the PM2 group viruses (see above). The DJR MCP and M13-like RCRE are the only two proteins that are encoded in all these contigs although a few additional, uncharacterized proteins are encoded in most of them. One of these contigs belongs to a previously isolated *Cellulophaga* phage phi 48:2 [74], several are assigned to bacterial genomes from the phylum Bacteroidetes and might represent prophages, and the majority are unaffiliated metagenomic sequences. Some of these sequences are flanked with repeats and therefore might comprise complete phage genomes. Should that be the case, this group of bacteriophages includes some of the largest identified ssDNA viruses, up to 17 kb in size, second only to archaeal viruses of the family *Spiraviridae* [75].

## Discussion

The results of this work are not unexpected in the sense that they are fully compatible with the notion of accelerating expansion of the virosphere thanks to genome and metagenome mining efforts [76]. It seems that, with the advances of metagenomics, an exhaustive search for distant relatives of any known group of viruses or for completely new groups is bound to reveal previously unsuspected diversity. Here we expanded the previously limited diversity and host range of small dsDNA (and in the case of the FLiP group, ssDNA) viruses of prokaryotes with icosahedral virions composed of DJR MCP. These findings restore the balance between viruses of prokaryotes and eukaryotes in the DJR MCP supergroup by demonstrating the wide spread of these viruses in prokaryotes. Although these viruses appear to be less abundant than the HK97 supergroup, their diversity, association with various hosts and presence in many environments revealed by the present analysis suggest that they comprise a substantial component of the prokaryotic virosphere and might be important ecological agents. While this manuscript was in review, a study describing a new family of tailless bacteriophages encoding a DJR MCP and denoted “Autolykiviridae” has been published [77]. The autolykiviruses have been identified as the principal killers of the Vibrionaceae bacteria in the ocean, indicating a much greater ecological impact of tailless bacteriophages than previously suspected. Notably, all members of this putative new family belong to the PM2 group, one of the 6 groups of DJR MCP viruses of prokaryotes described here. In the phylogenetic tree of the MCP for the PM2 group, the autolykiviruses form one tight clade among several (Additional file 3), emphasizing the diversity of the prokaryotic DJR MCP viruses of which the new family is but a small part. Taken together, these findings reveal an unexpectedly wide spread of the DJR MCP class of viruses in the biosphere the full extent of which remains to be assessed.

The search for new viruses described here was performed using a straightforward approach, namely, searching the genomic and metagenomics databases for MCP homologs. It should be emphasized, however, that using the most sensitive and specific of the available database search approaches is critical for the success of such efforts. The majority of the putative viruses and proviruses that we describe here could be identified only when a manually curated alignment of MCP was used as the query for database search. Furthermore, many other proteins of the identified viruses are also highly diverged, making manual curation a must for robust genome analysis.

The putative viruses identified here with the single MCP probe show consistency in terms of genome size: all have some of the smallest genomes among dsDNA viruses, roughly, between 7 and 18 kb. These elements also encode varying assortments of proteins from a characteristic virus gene pool, the most prominent being the packaging ATPase of the FtsK-HerA superfamily. This consistency of genomic features suggests that the prokaryotic DJR MCP-encoding viruses occupy a distinct part of the virosphere. However, the other side of the coin is the extreme plasticity of the gene repertoires of these viruses. Not a single gene other than MCP that is present by design of our search protocol is shared by all genomes. Instead, within each of the defined virus groups, proteins from the replication, integration and lysis modules are recurrently replaced with functionally equivalent counterparts. Even the packaging ATPase that is one of the most stable functional partners of the DJR MCP is missing in the putative viruses of the Odin and FLiP groups, suggesting a distinct packaging mechanism. The viruses of the newly discovered Odin group have the smallest genomes in the DJR MCP supergroup, comparable in size to the genomes of the smallest dsDNA viruses of eukaryotes (polyoma/papillomaviruses) and ssDNA viruses with single jelly roll MCPs that lack a dedicated virus-encoded packaging ATPase. Many of these viruses appear to assemble the capsid around the viral genome [78, 79] instead of packaging the DNA into a preformed, empty capsid in an ATP-dependent fashion as dsDNA viruses with larger genomes do [80]. A similar mechanism might be operative in the viruses of the Odin group as well as the FLiP group. More generally, the present findings emphasize the enormous evolutionary plasticity of viruses that can completely change the gene repertoire while retaining the same capsid structure and similar genome size. Parallel findings have been reported previously as a result of a search for eukaryotic DJR MCP viruses resembling the polinton class transposons [72, 81], suggesting that such plasticity is a general, still under-appreciated trend in the evolution of the virus world. It is our hope that the present analysis stimulates experimental characterization of some of the viruses identified here which will shed light on virus biology.

## Conclusions

The findings reported here continue the general trend of metagenomic discovery whereby searches of sequence databases for new viruses employing powerful computational methods expand the diversity of (putative) viruses far beyond that established by traditional methods for virus isolation. It is not uncommon that groups of viruses that appeared relatively rare and minor, possibly, due to biases caused by difficulty of host cultivation, become comparable, in diversity and abundance, to previously identified major groups. Here, such a dramatic expansion is reported for the prokaryotic viruses encoding DJR MCP. The results suggest that these viruses that are the apparent ancestors of the most common dsDNA viruses of eukaryotes [21, 30] are important players in the prokaryotic virosphere as well. Furthermore, these small viruses show striking evolutionary plasticity such that not a single gene except for that encoding the MCP is shared by all of them.

## Additional files

Additional file 1: HHpred search results of conserved proteins of Odin and STIV groups. (ZIP 648 kb)

Additional file 2: Odin group genome maps. (PPTX 127 kb)

Additional file 3: PM2 group MCP tree. (PPTX 70 kb)

Additional file 4: Genbank proteins identified as PM2 group MCP. (ZIP 5 kb)

Additional file 5: DNA polymerase tree. (PPTX 86 kb)

Additional file 6: STIV group genome maps. (PPTX 216 kb)

Additional file 7: Bam35-Toil group genome maps. (PPTX 193 kb)

Additional file 8: PRD1 group MCP tree. (PPTX 36 kb)
