# Supplementary material for: Vast diversity of prokaryotic virus genomes encoding double jelly-roll major capsid proteins uncovered by genomic and metagenomic sequence analysis
Source: Virol J. 2018 Apr 10;15:67. doi: 10.1186/s12985-018-0974-y (PMC5894146; doi:10.1186/s12985-018-0974-y)
Supplement: Supplementary file 2 — Odin group genome maps. (PPTX 127 kb) [file 12985_2018_974_MOESM2_ESM.pptx]

## Slide 1
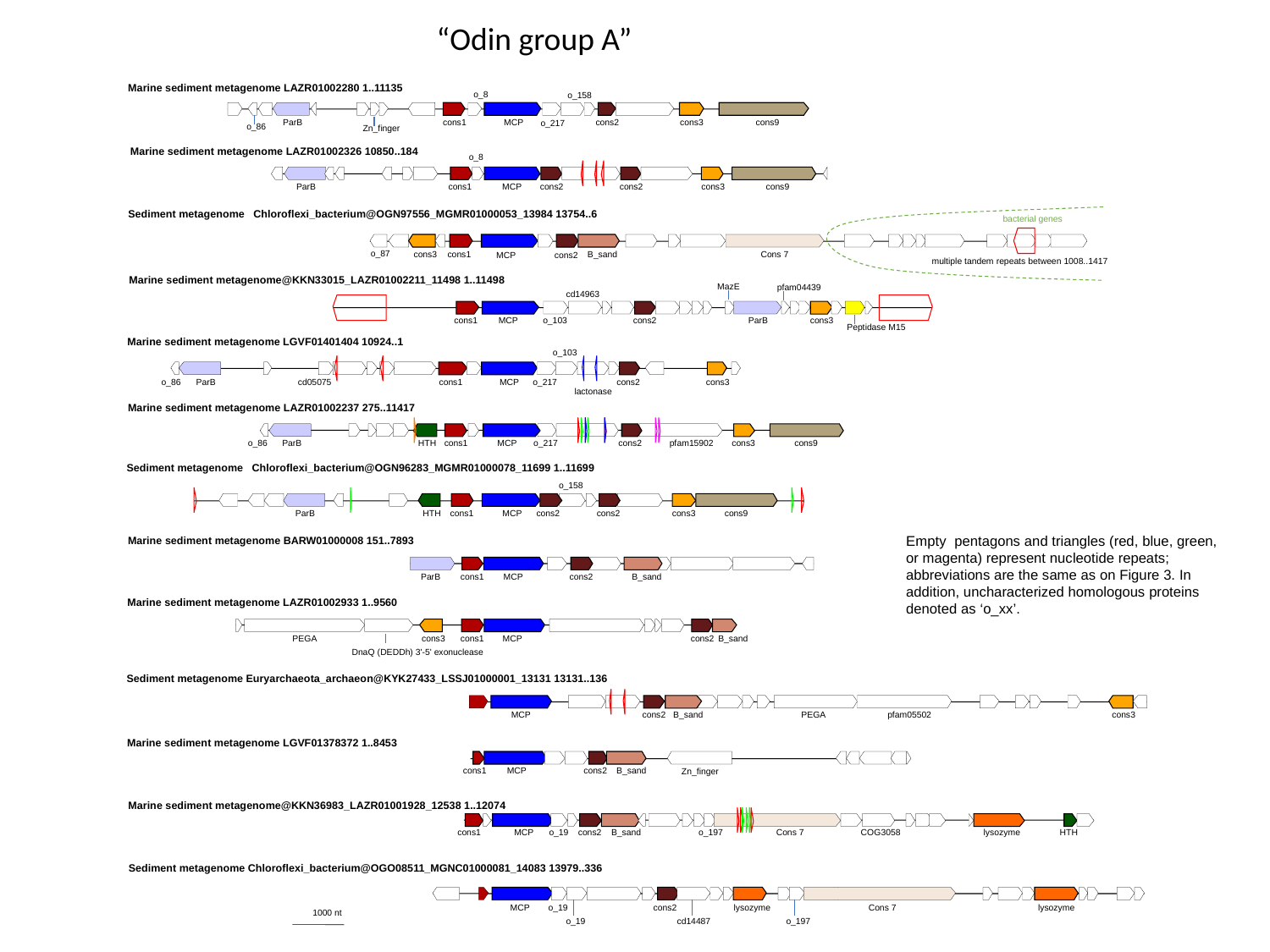

“Odin group A”
Marine sediment metagenome LAZR01002280 1..11135
o_8
o_158
ParB
cons1
MCP
cons2
cons3
cons9
o_217
o_86
Zn_finger
 Marine sediment metagenome LAZR01002326 10850..184
o_8
ParB
cons1
MCP
cons2
cons2
cons3
cons9
bacterial genes
o_87
cons3
cons1
B_sand
cons2
MCP
multiple tandem repeats between 1008..1417
Cons 7
Sediment metagenome Chloroflexi_bacterium@OGN97556_MGMR01000053_13984 13754..6
Marine sediment metagenome@KKN33015_LAZR01002211_11498 1..11498
MazE
pfam04439
cd14963
cons1
MCP
o_103
cons2
ParB
cons3
Peptidase M15
Marine sediment metagenome LGVF01401404 10924..1
o_103
o_86
ParB
cd05075
cons1
MCP
o_217
cons2
cons3
lactonase
Marine sediment metagenome LAZR01002237 275..11417
o_86
ParB
HTH
cons1
MCP
o_217
cons2
pfam15902
cons3
cons9
Sediment metagenome Chloroflexi_bacterium@OGN96283_MGMR01000078_11699 1..11699
o_158
ParB
HTH
cons1
MCP
cons2
cons2
cons3
cons9
Empty pentagons and triangles (red, blue, green, or magenta) represent nucleotide repeats; abbreviations are the same as on Figure 3. In addition, uncharacterized homologous proteins denoted as ‘o_xx’.
Marine sediment metagenome BARW01000008 151..7893
ParB
cons1
MCP
cons2
B_sand
Marine sediment metagenome LAZR01002933 1..9560
PEGA
cons3
cons1
MCP
cons2
B_sand
DnaQ (DEDDh) 3'-5' exonuclease
Sediment metagenome Euryarchaeota_archaeon@KYK27433_LSSJ01000001_13131 13131..136
MCP
cons2
B_sand
PEGA
pfam05502
cons3
Marine sediment metagenome LGVF01378372 1..8453
cons1
MCP
cons2
B_sand
Zn_finger
Marine sediment metagenome@KKN36983_LAZR01001928_12538 1..12074
cons1
MCP
o_19
cons2
B_sand
o_197
Cons 7
COG3058
lysozyme
HTH
Sediment metagenome Chloroflexi_bacterium@OGO08511_MGNC01000081_14083 13979..336
MCP
o_19
cons2
lysozyme
Cons 7
lysozyme
o_19
cd14487
o_197
1000 nt

## Slide 2
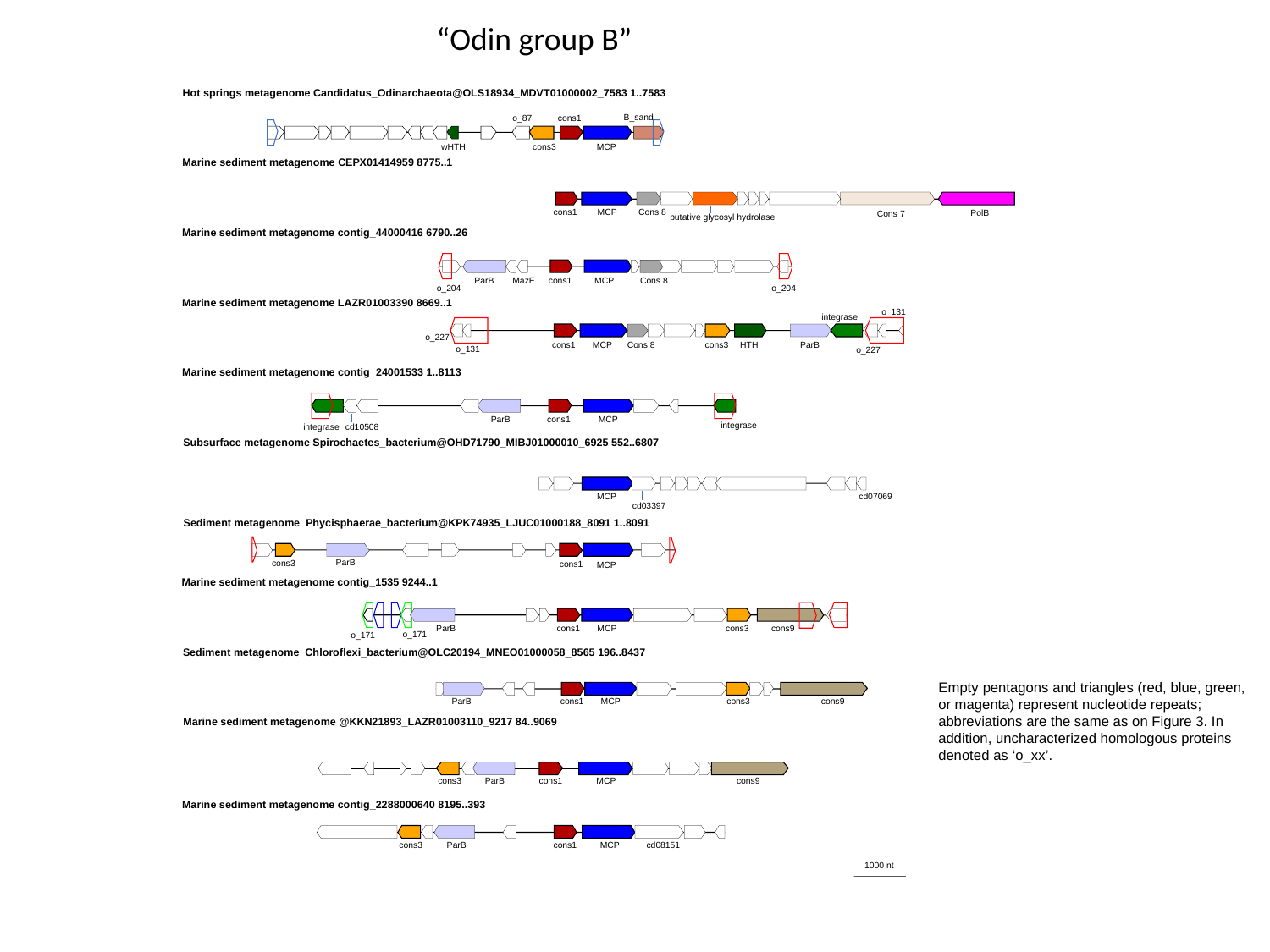

“Odin group B”
Hot springs metagenome Candidatus_Odinarchaeota@OLS18934_MDVT01000002_7583 1..7583
Marine sediment metagenome CEPX01414959 8775..1
Marine sediment metagenome contig_44000416 6790..26
Marine sediment metagenome LAZR01003390 8669..1
Marine sediment metagenome contig_24001533 1..8113
Subsurface metagenome Spirochaetes_bacterium@OHD71790_MIBJ01000010_6925 552..6807
Sediment metagenome Phycisphaerae_bacterium@KPK74935_LJUC01000188_8091 1..8091
Marine sediment metagenome contig_1535 9244..1
Sediment metagenome Chloroflexi_bacterium@OLC20194_MNEO01000058_8565 196..8437
Marine sediment metagenome @KKN21893_LAZR01003110_9217 84..9069
Marine sediment metagenome contig_2288000640 8195..393
B_sand
o_87
cons1
wHTH
cons3
MCP
cons1
MCP
Cons 8
PolB
putative glycosyl hydrolase
ParB
MazE
cons1
MCP
Cons 8
o_204
o_204
o_131
integrase
o_227
cons1
MCP
Cons 8
cons3
HTH
ParB
o_131
o_227
ParB
cons1
MCP
integrase
cd10508
integrase
MCP
cd07069
cd03397
ParB
cons3
cons1
MCP
ParB
cons1
MCP
cons3
cons9
o_171
o_171
ParB
cons1
MCP
cons3
cons9
cons3
ParB
cons1
MCP
cons9
cons3
ParB
cons1
MCP
cd08151
Cons 7
Empty pentagons and triangles (red, blue, green, or magenta) represent nucleotide repeats; abbreviations are the same as on Figure 3. In addition, uncharacterized homologous proteins denoted as ‘o_xx’.
1000 nt
