# Supplementary material for: Vast diversity of prokaryotic virus genomes encoding double jelly-roll major capsid proteins uncovered by genomic and metagenomic sequence analysis
Source: Virol J. 2018 Apr 10;15:67. doi: 10.1186/s12985-018-0974-y (PMC5894146; doi:10.1186/s12985-018-0974-y)
Supplement: Supplementary file 3 — PM2 group MCP tree. (PPTX 70 kb) [file 12985_2018_974_MOESM3_ESM.pptx]

## Slide 1
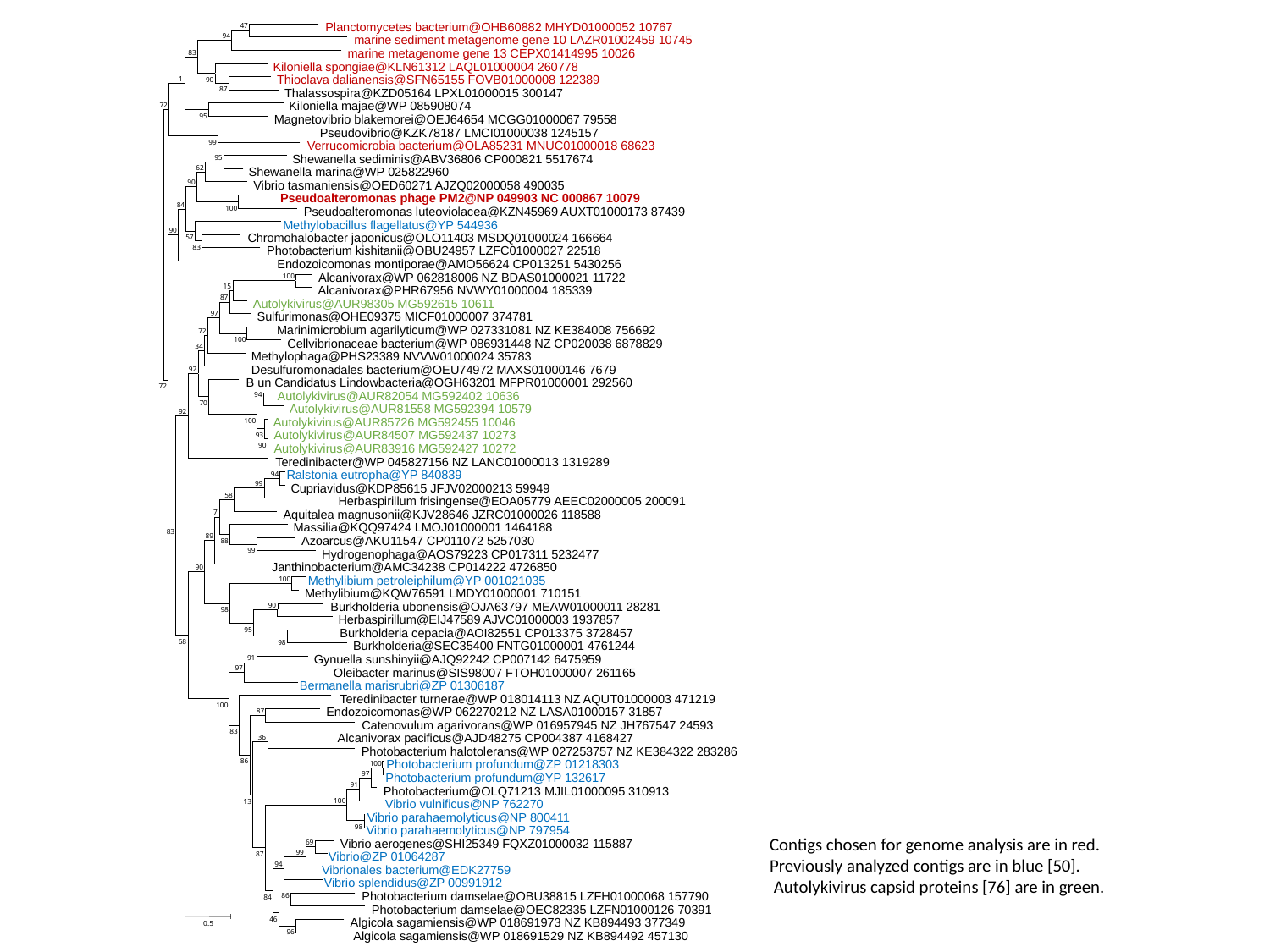

Planctomycetes bacterium@OHB60882 MHYD01000052 10767
47
94
 marine sediment metagenome gene 10 LAZR01002459 10745
 marine metagenome gene 13 CEPX01414995 10026
83
 Kiloniella spongiae@KLN61312 LAQL01000004 260778
 Thioclava dalianensis@SFN65155 FOVB01000008 122389
1
90
87
 Thalassospira@KZD05164 LPXL01000015 300147
 Kiloniella majae@WP 085908074
72
95
 Magnetovibrio blakemorei@OEJ64654 MCGG01000067 79558
 Pseudovibrio@KZK78187 LMCI01000038 1245157
99
 Verrucomicrobia bacterium@OLA85231 MNUC01000018 68623
 Shewanella sediminis@ABV36806 CP000821 5517674
95
62
 Shewanella marina@WP 025822960
 Vibrio tasmaniensis@OED60271 AJZQ02000058 490035
90
 Pseudoalteromonas phage PM2@NP 049903 NC 000867 10079
84
100
 Pseudoalteromonas luteoviolacea@KZN45969 AUXT01000173 87439
Methylobacillus flagellatus@YP 544936
90
 Chromohalobacter japonicus@OLO11403 MSDQ01000024 166664
57
83
 Photobacterium kishitanii@OBU24957 LZFC01000027 22518
 Endozoicomonas montiporae@AMO56624 CP013251 5430256
 Alcanivorax@WP 062818006 NZ BDAS01000021 11722
100
15
 Alcanivorax@PHR67956 NVWY01000004 185339
87
 Autolykivirus@AUR98305 MG592615 10611
97
 Sulfurimonas@OHE09375 MICF01000007 374781
 Marinimicrobium agarilyticum@WP 027331081 NZ KE384008 756692
72
100
 Cellvibrionaceae bacterium@WP 086931448 NZ CP020038 6878829
34
 Methylophaga@PHS23389 NVVW01000024 35783
 Desulfuromonadales bacterium@OEU74972 MAXS01000146 7679
92
 B un Candidatus Lindowbacteria@OGH63201 MFPR01000001 292560
72
 Autolykivirus@AUR82054 MG592402 10636
94
70
 Autolykivirus@AUR81558 MG592394 10579
92
 Autolykivirus@AUR85726 MG592455 10046
100
 Autolykivirus@AUR84507 MG592437 10273
93
90
 Autolykivirus@AUR83916 MG592427 10272
 Teredinibacter@WP 045827156 NZ LANC01000013 1319289
Ralstonia eutropha@YP 840839
94
99
 Cupriavidus@KDP85615 JFJV02000213 59949
58
 Herbaspirillum frisingense@EOA05779 AEEC02000005 200091
 Aquitalea magnusonii@KJV28646 JZRC01000026 118588
7
 Massilia@KQQ97424 LMOJ01000001 1464188
83
89
 Azoarcus@AKU11547 CP011072 5257030
88
99
 Hydrogenophaga@AOS79223 CP017311 5232477
 Janthinobacterium@AMC34238 CP014222 4726850
90
Methylibium petroleiphilum@YP 001021035
100
 Methylibium@KQW76591 LMDY01000001 710151
 Burkholderia ubonensis@OJA63797 MEAW01000011 28281
90
98
 Herbaspirillum@EIJ47589 AJVC01000003 1937857
95
 Burkholderia cepacia@AOI82551 CP013375 3728457
68
98
 Burkholderia@SEC35400 FNTG01000001 4761244
 Gynuella sunshinyii@AJQ92242 CP007142 6475959
91
97
 Oleibacter marinus@SIS98007 FTOH01000007 261165
Bermanella marisrubri@ZP 01306187
 Teredinibacter turnerae@WP 018014113 NZ AQUT01000003 471219
100
 Endozoicomonas@WP 062270212 NZ LASA01000157 31857
87
 Catenovulum agarivorans@WP 016957945 NZ JH767547 24593
83
 Alcanivorax pacificus@AJD48275 CP004387 4168427
36
 Photobacterium halotolerans@WP 027253757 NZ KE384322 283286
86
Photobacterium profundum@ZP 01218303
100
97
Photobacterium profundum@YP 132617
91
 Photobacterium@OLQ71213 MJIL01000095 310913
100
Vibrio vulnificus@NP 762270
13
Vibrio parahaemolyticus@NP 800411
98
Vibrio parahaemolyticus@NP 797954
 Vibrio aerogenes@SHI25349 FQXZ01000032 115887
69
99
Vibrio@ZP 01064287
87
94
Vibrionales bacterium@EDK27759
Vibrio splendidus@ZP 00991912
 Photobacterium damselae@OBU38815 LZFH01000068 157790
86
84
 Photobacterium damselae@OEC82335 LZFN01000126 70391
46
 Algicola sagamiensis@WP 018691973 NZ KB894493 377349
0.5
96
 Algicola sagamiensis@WP 018691529 NZ KB894492 457130
Contigs chosen for genome analysis are in red.
Previously analyzed contigs are in blue [50].
 Autolykivirus capsid proteins [76] are in green.
