# Supplementary material for: Vast diversity of prokaryotic virus genomes encoding double jelly-roll major capsid proteins uncovered by genomic and metagenomic sequence analysis
Source: Virol J. 2018 Apr 10;15:67. doi: 10.1186/s12985-018-0974-y (PMC5894146; doi:10.1186/s12985-018-0974-y)
Supplement: Supplementary file 5 — DNA polymerase tree. (PPTX 86 kb) [file 12985_2018_974_MOESM5_ESM.pptx]

## Slide 1
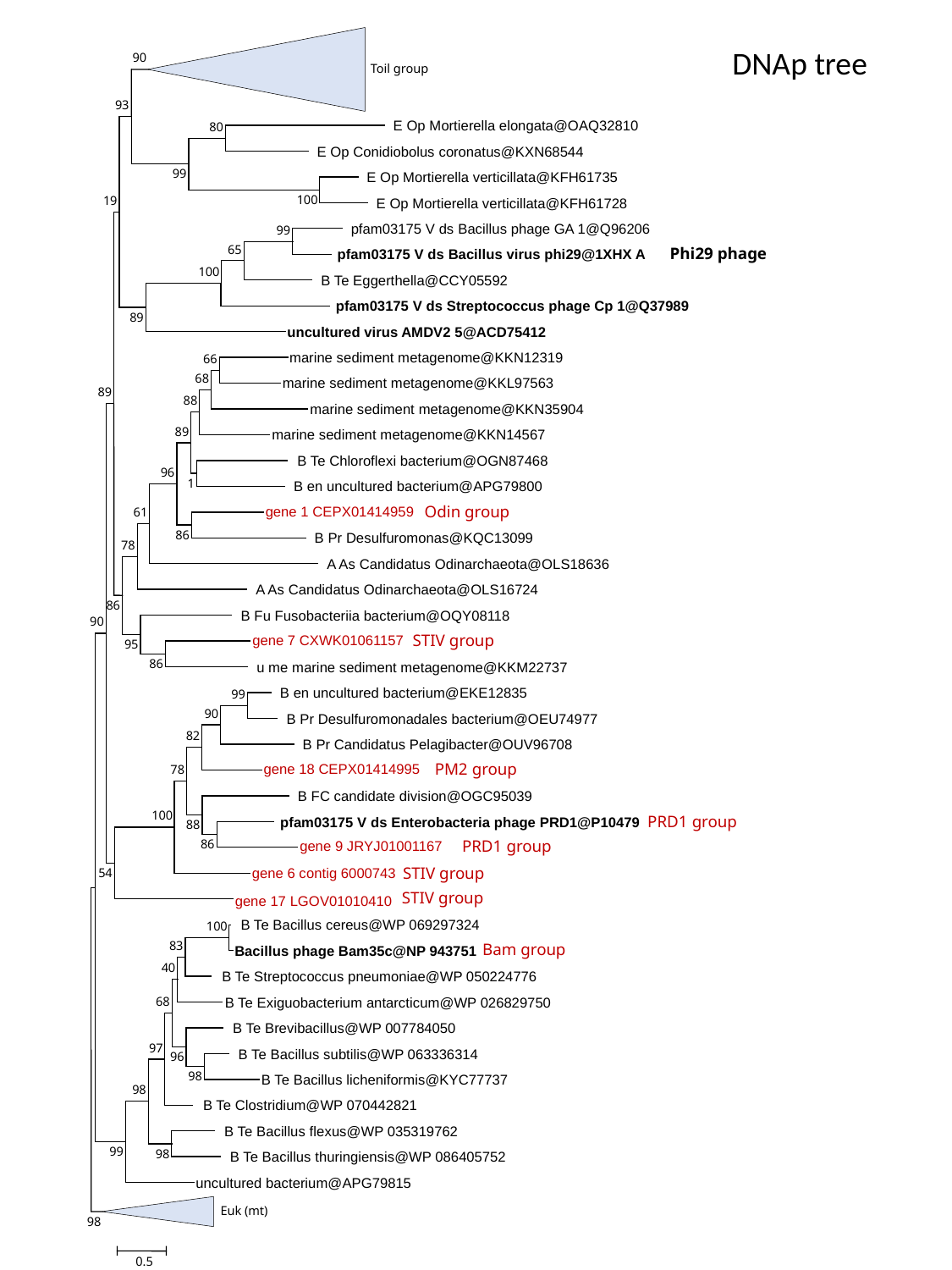

DNAp tree
90
 Toil group
93
 E Op Mortierella elongata@OAQ32810
80
 E Op Conidiobolus coronatus@KXN68544
99
 E Op Mortierella verticillata@KFH61735
100
19
 E Op Mortierella verticillata@KFH61728
 pfam03175 V ds Bacillus phage GA 1@Q96206
99
65
Phi29 phage
 pfam03175 V ds Bacillus virus phi29@1XHX A
100
 B Te Eggerthella@CCY05592
 pfam03175 V ds Streptococcus phage Cp 1@Q37989
89
uncultured virus AMDV2 5@ACD75412
marine sediment metagenome@KKN12319
66
68
marine sediment metagenome@KKL97563
89
88
marine sediment metagenome@KKN35904
89
marine sediment metagenome@KKN14567
 B Te Chloroflexi bacterium@OGN87468
96
1
 B en uncultured bacterium@APG79800
Odin group
gene 1 CEPX01414959
61
86
 B Pr Desulfuromonas@KQC13099
78
 A As Candidatus Odinarchaeota@OLS18636
 A As Candidatus Odinarchaeota@OLS16724
86
 B Fu Fusobacteriia bacterium@OQY08118
90
STIV group
gene 7 CXWK01061157
95
86
 u me marine sediment metagenome@KKM22737
 B en uncultured bacterium@EKE12835
99
90
 B Pr Desulfuromonadales bacterium@OEU74977
82
 B Pr Candidatus Pelagibacter@OUV96708
PM2 group
gene 18 CEPX01414995
78
 B FC candidate division@OGC95039
100
PRD1 group
 pfam03175 V ds Enterobacteria phage PRD1@P10479
88
86
PRD1 group
gene 9 JRYJ01001167
STIV group
gene 6 contig 6000743
54
STIV group
gene 17 LGOV01010410
 B Te Bacillus cereus@WP 069297324
100
83
Bam group
Bacillus phage Bam35c@NP 943751
40
 B Te Streptococcus pneumoniae@WP 050224776
B Te Exiguobacterium antarcticum@WP 026829750
68
 B Te Brevibacillus@WP 007784050
97
 B Te Bacillus subtilis@WP 063336314
96
98
B Te Bacillus licheniformis@KYC77737
98
 B Te Clostridium@WP 070442821
 B Te Bacillus flexus@WP 035319762
99
98
 B Te Bacillus thuringiensis@WP 086405752
uncultured bacterium@APG79815
 Euk (mt)
98
0.5

## Slide 2
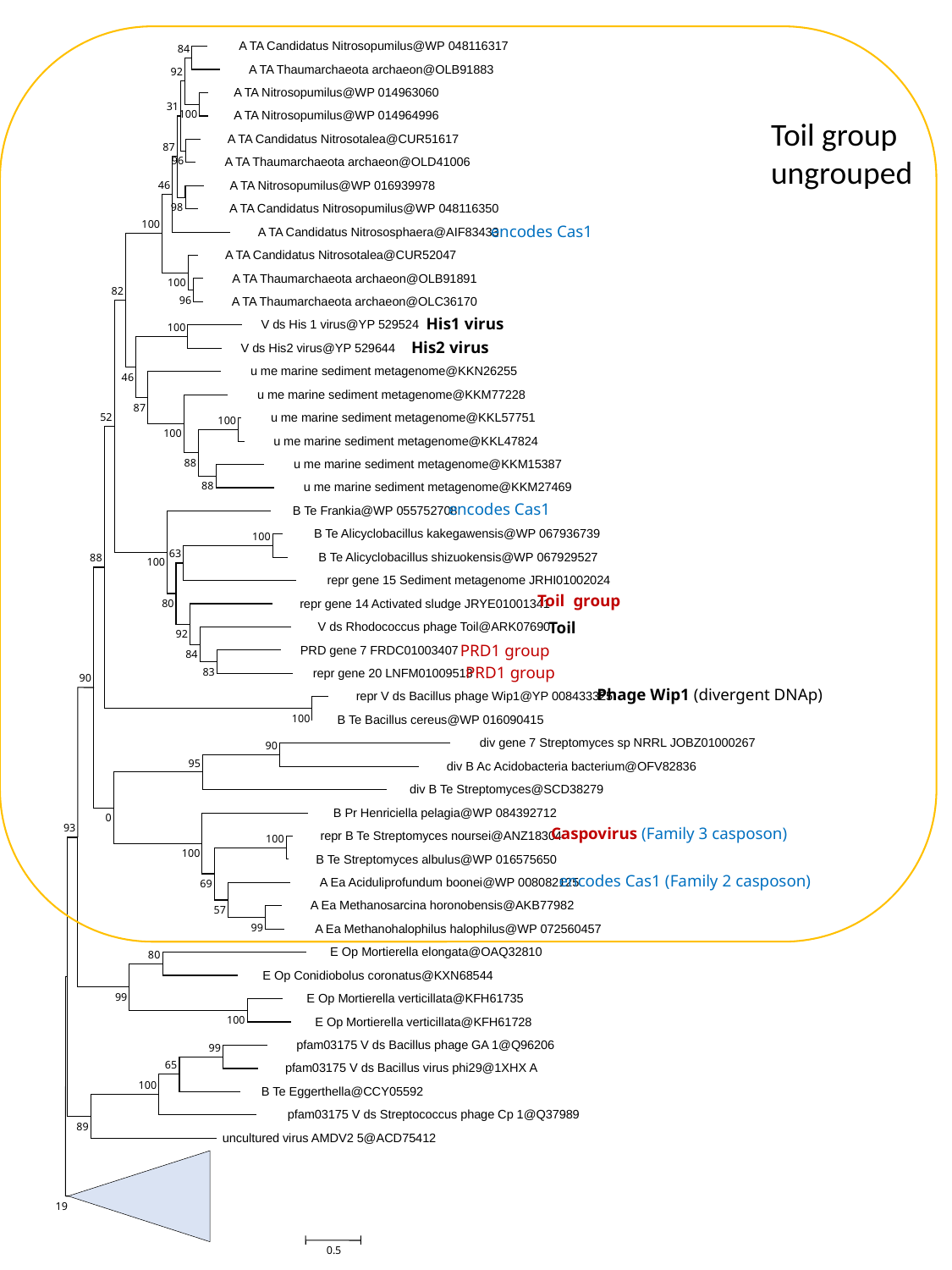

A TA Candidatus Nitrosopumilus@WP 048116317
84
 A TA Thaumarchaeota archaeon@OLB91883
92
 A TA Nitrosopumilus@WP 014963060
31
100
 A TA Nitrosopumilus@WP 014964996
Toil group ungrouped
 A TA Candidatus Nitrosotalea@CUR51617
87
96
 A TA Thaumarchaeota archaeon@OLD41006
 A TA Nitrosopumilus@WP 016939978
46
98
 A TA Candidatus Nitrosopumilus@WP 048116350
100
encodes Cas1
 A TA Candidatus Nitrososphaera@AIF83433
 A TA Candidatus Nitrosotalea@CUR52047
 A TA Thaumarchaeota archaeon@OLB91891
100
82
96
 A TA Thaumarchaeota archaeon@OLC36170
His1 virus
 V ds His 1 virus@YP 529524
100
His2 virus
 V ds His2 virus@YP 529644
 u me marine sediment metagenome@KKN26255
46
 u me marine sediment metagenome@KKM77228
87
 u me marine sediment metagenome@KKL57751
52
100
100
 u me marine sediment metagenome@KKL47824
88
 u me marine sediment metagenome@KKM15387
88
 u me marine sediment metagenome@KKM27469
encodes Cas1
 B Te Frankia@WP 055752708
 B Te Alicyclobacillus kakegawensis@WP 067936739
100
63
 B Te Alicyclobacillus shizuokensis@WP 067929527
88
100
 repr gene 15 Sediment metagenome JRHI01002024
Toil group
 repr gene 14 Activated sludge JRYE01001341
80
Toil
 V ds Rhodococcus phage Toil@ARK07690
92
PRD1 group
 PRD gene 7 FRDC01003407
84
PRD1 group
83
 repr gene 20 LNFM01009513
90
Phage Wip1 (divergent DNAp)
 repr V ds Bacillus phage Wip1@YP 008433325
100
 B Te Bacillus cereus@WP 016090415
 div gene 7 Streptomyces sp NRRL JOBZ01000267
90
95
 div B Ac Acidobacteria bacterium@OFV82836
 div B Te Streptomyces@SCD38279
 B Pr Henriciella pelagia@WP 084392712
0
93
Caspovirus (Family 3 casposon)
 repr B Te Streptomyces noursei@ANZ18304
100
100
 B Te Streptomyces albulus@WP 016575650
encodes Cas1 (Family 2 casposon)
 A Ea Aciduliprofundum boonei@WP 008082125
69
 A Ea Methanosarcina horonobensis@AKB77982
57
99
 A Ea Methanohalophilus halophilus@WP 072560457
 E Op Mortierella elongata@OAQ32810
80
 E Op Conidiobolus coronatus@KXN68544
99
 E Op Mortierella verticillata@KFH61735
100
 E Op Mortierella verticillata@KFH61728
 pfam03175 V ds Bacillus phage GA 1@Q96206
99
65
 pfam03175 V ds Bacillus virus phi29@1XHX A
100
 B Te Eggerthella@CCY05592
 pfam03175 V ds Streptococcus phage Cp 1@Q37989
89
 uncultured virus AMDV2 5@ACD75412
19
0.5
