# Supplementary material for: Vast diversity of prokaryotic virus genomes encoding double jelly-roll major capsid proteins uncovered by genomic and metagenomic sequence analysis
Source: Virol J. 2018 Apr 10;15:67. doi: 10.1186/s12985-018-0974-y (PMC5894146; doi:10.1186/s12985-018-0974-y)
Supplement: Supplementary file 6 — STIV group genome maps. (PPTX 216 kb) [file 12985_2018_974_MOESM6_ESM.pptx]

## Slide 1
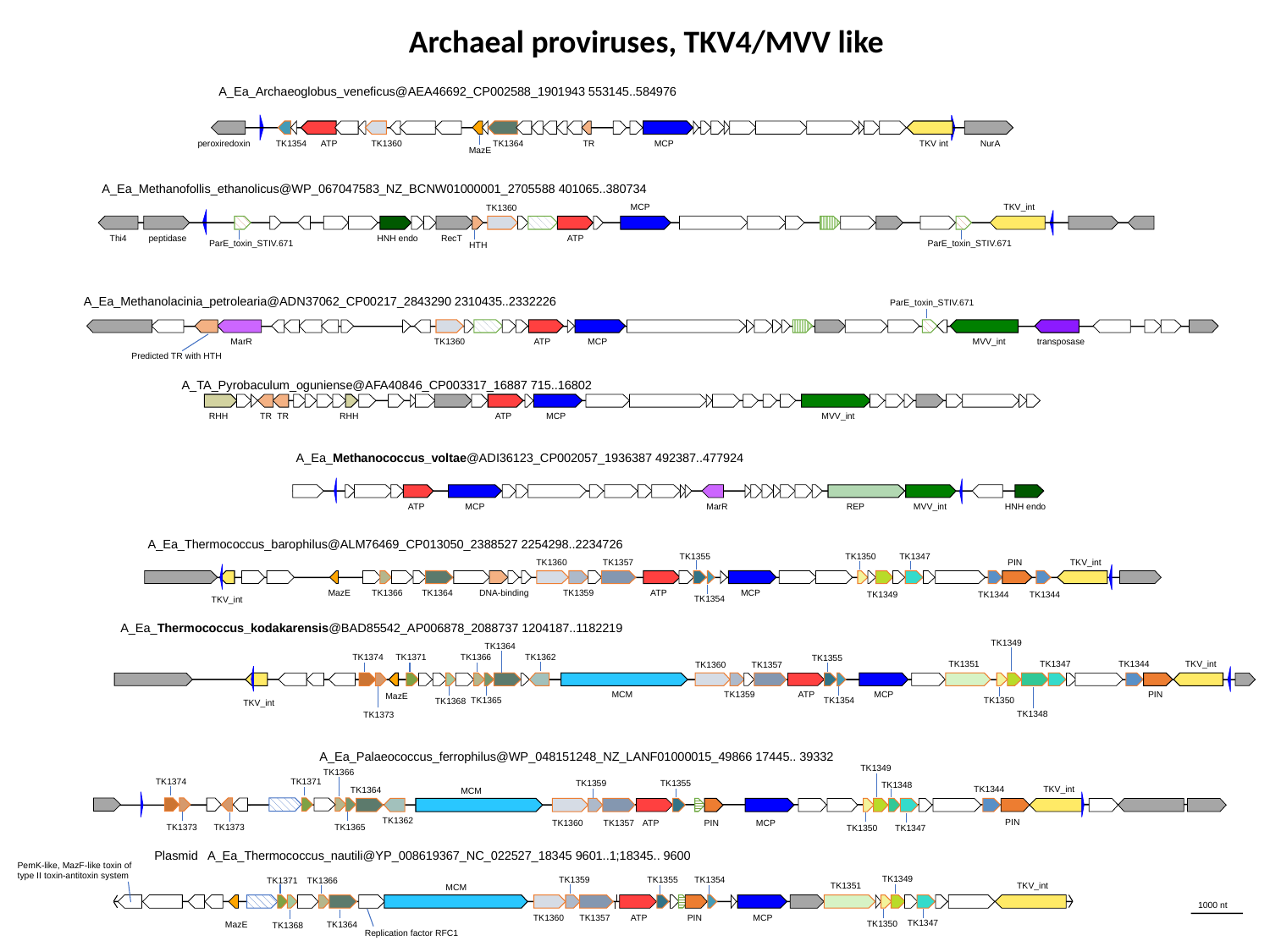

Archaeal proviruses, TKV4/MVV like
A_Ea_Archaeoglobus_veneficus@AEA46692_CP002588_1901943 553145..584976
peroxiredoxin
TK1354
ATP
TK1360
TK1364
TR
MCP
TKV int
NurA
MazE
A_Ea_Methanofollis_ethanolicus@WP_067047583_NZ_BCNW01000001_2705588 401065..380734
MCP
TKV_int
TK1360
Thi4
peptidase
HNH endo
RecT
ATP
ParE_toxin_STIV.671
ParE_toxin_STIV.671
HTH
A_Ea_Methanolacinia_petrolearia@ADN37062_CP00217_2843290 2310435..2332226
ParE_toxin_STIV.671
MarR
TK1360
ATP
MCP
MVV_int
transposase
Predicted TR with HTH
A_TA_Pyrobaculum_oguniense@AFA40846_CP003317_16887 715..16802
RHH
TR
TR
RHH
ATP
MCP
MVV_int
A_Ea_Methanococcus_voltae@ADI36123_CP002057_1936387 492387..477924
ATP
MCP
MarR
REP
MVV_int
HNH endo
A_Ea_Thermococcus_barophilus@ALM76469_CP013050_2388527 2254298..2234726
TK1350
TK1347
TK1355
TK1360
TK1357
PIN
TKV_int
MazE
TK1366
TK1364
DNA-binding
TK1359
ATP
MCP
TK1349
TK1344
TK1344
TK1354
TKV_int
A_Ea_Thermococcus_kodakarensis@BAD85542_AP006878_2088737 1204187..1182219
TK1349
TK1364
TK1374
TK1371
TK1366
TK1362
TK1355
TK1351
TK1347
TK1344
TKV_int
TK1360
TK1357
MCM
TK1359
ATP
MCP
PIN
MazE
TK1365
TK1350
TK1354
TK1368
TKV_int
TK1348
TK1373
A_Ea_Palaeococcus_ferrophilus@WP_048151248_NZ_LANF01000015_49866 17445.. 39332
TK1349
TK1366
TK1374
TK1371
TK1359
TK1355
TK1348
TK1344
TKV_int
TK1364
MCM
TK1362
PIN
TK1360
TK1357
ATP
PIN
MCP
TK1373
TK1373
TK1365
TK1350
TK1347
Plasmid A_Ea_Thermococcus_nautili@YP_008619367_NC_022527_18345 9601..1;18345.. 9600
PemK-like, MazF-like toxin of type II toxin-antitoxin system
TK1349
TK1359
TK1355
TK1354
TK1371
TK1366
TK1351
TKV_int
MCM
1000 nt
TK1360
TK1357
ATP
PIN
MCP
TK1347
TK1350
MazE
TK1364
TK1368
Replication factor RFC1

## Slide 2
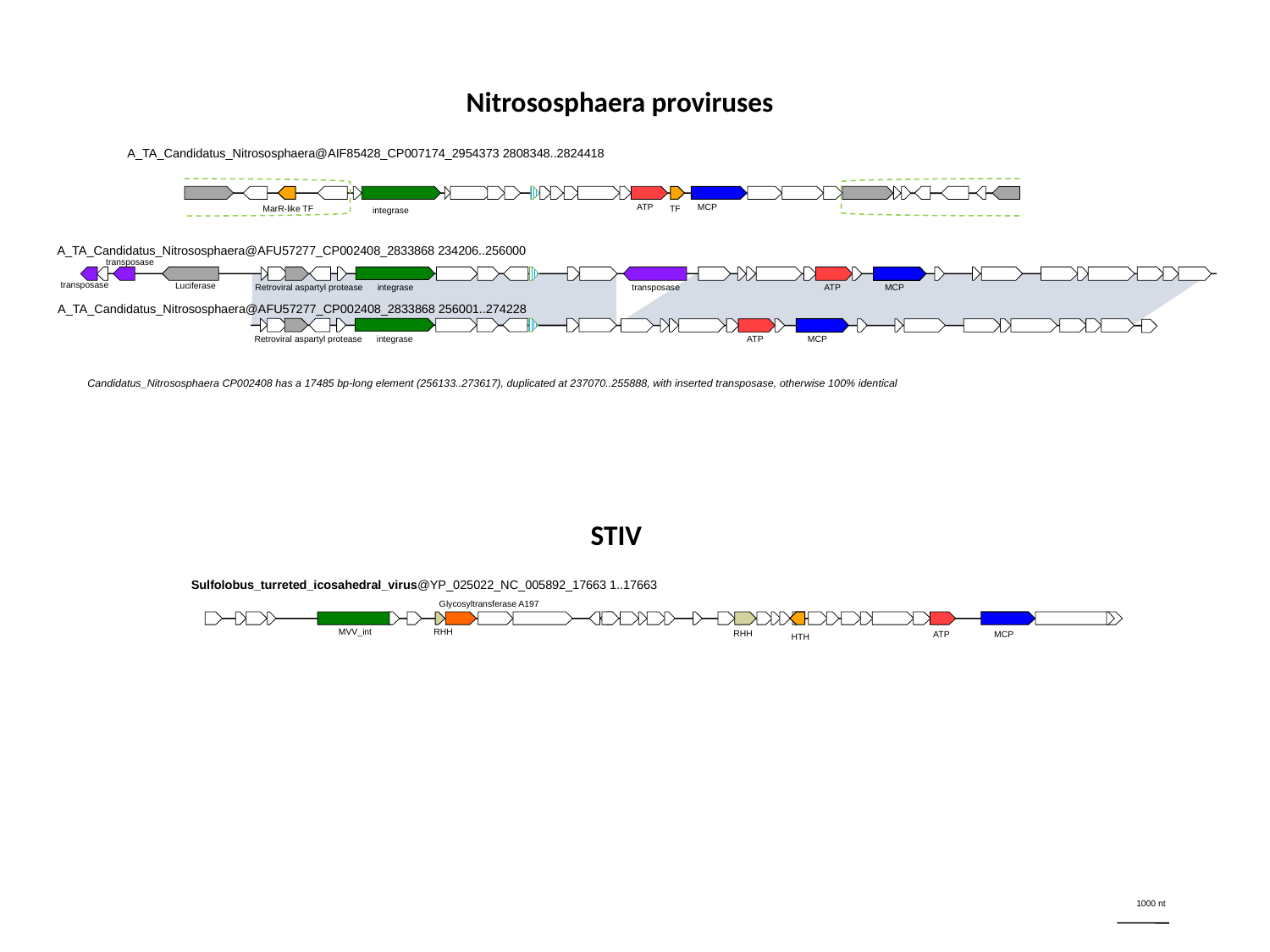

Nitrososphaera proviruses
A_TA_Candidatus_Nitrososphaera@AIF85428_CP007174_2954373 2808348..2824418
MCP
ATP
MarR-like TF
TF
integrase
A_TA_Candidatus_Nitrososphaera@AFU57277_CP002408_2833868 234206..256000
transposase
transposase
Retroviral aspartyl protease
integrase
ATP
MCP
transposase
Luciferase
A_TA_Candidatus_Nitrososphaera@AFU57277_CP002408_2833868 256001..274228
Retroviral aspartyl protease
integrase
ATP
MCP
Candidatus_Nitrososphaera CP002408 has a 17485 bp-long element (256133..273617), duplicated at 237070..255888, with inserted transposase, otherwise 100% identical
STIV
Sulfolobus_turreted_icosahedral_virus@YP_025022_NC_005892_17663 1..17663
Glycosyltransferase A197
RHH
MVV_int
RHH
MCP
ATP
HTH
1000 nt

## Slide 3
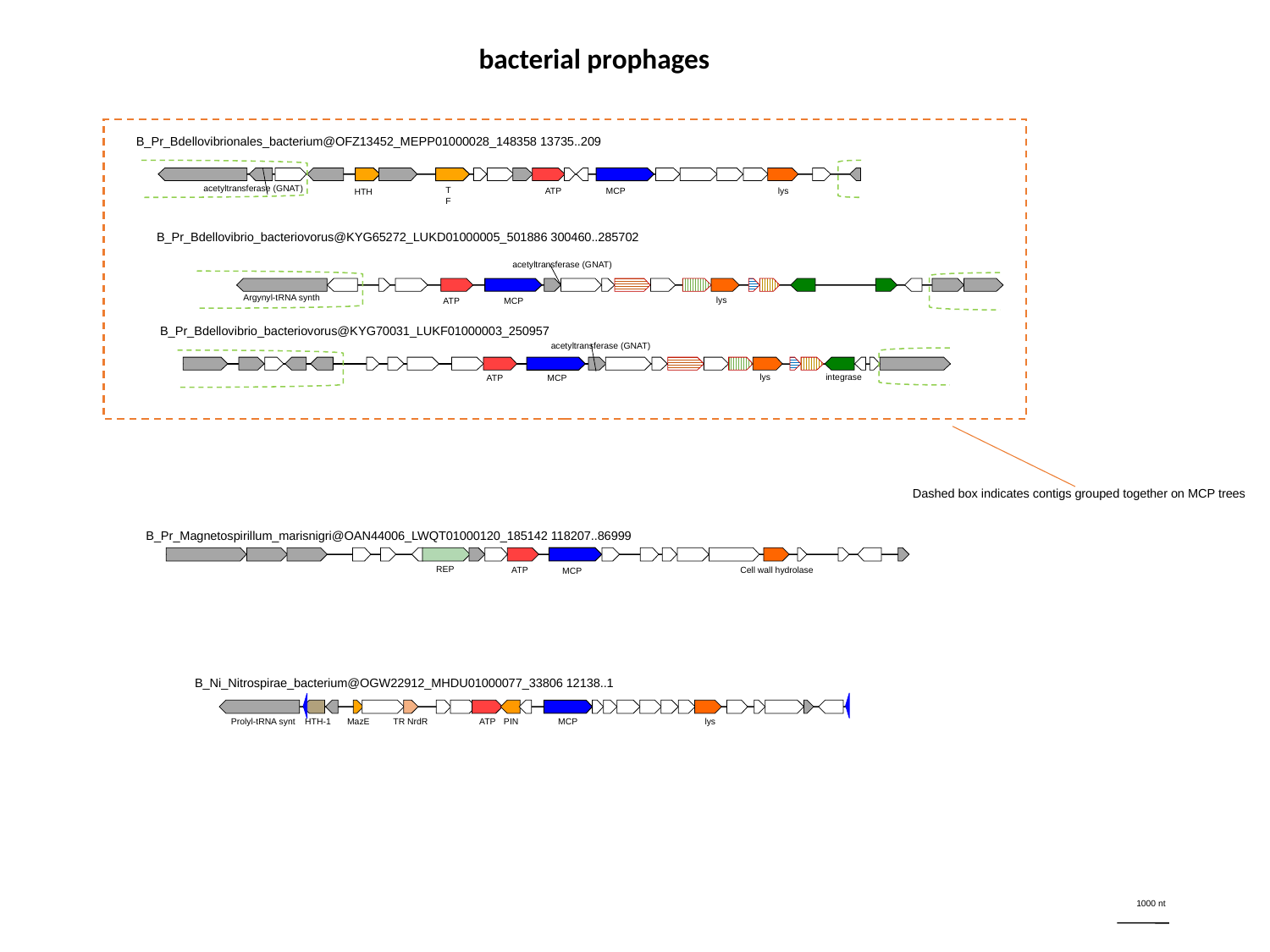

bacterial prophages
B_Pr_Bdellovibrionales_bacterium@OFZ13452_MEPP01000028_148358 13735..209
acetyltransferase (GNAT)
TF
MCP
lys
ATP
HTH
B_Pr_Bdellovibrio_bacteriovorus@KYG65272_LUKD01000005_501886 300460..285702
acetyltransferase (GNAT)
Argynyl-tRNA synth
lys
MCP
ATP
B_Pr_Bdellovibrio_bacteriovorus@KYG70031_LUKF01000003_250957
acetyltransferase (GNAT)
lys
integrase
MCP
ATP
Dashed box indicates contigs grouped together on MCP trees
B_Pr_Magnetospirillum_marisnigri@OAN44006_LWQT01000120_185142 118207..86999
REP
ATP
MCP
Cell wall hydrolase
B_Ni_Nitrospirae_bacterium@OGW22912_MHDU01000077_33806 12138..1
Prolyl-tRNA synt
HTH-1
MazE
TR NrdR
ATP
PIN
MCP
lys
1000 nt

## Slide 4
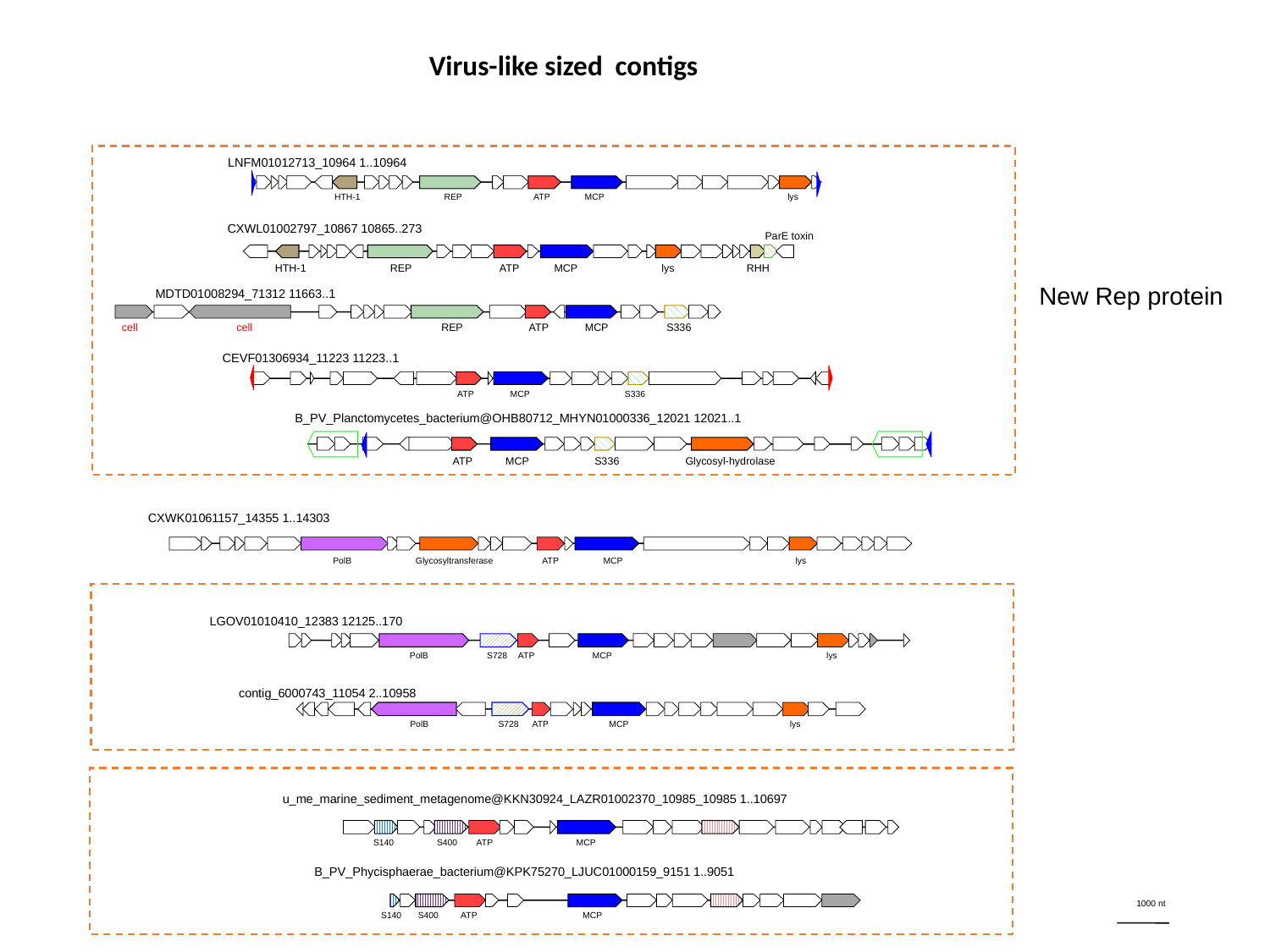

Virus-like sized contigs
LNFM01012713_10964 1..10964
HTH-1
REP
ATP
MCP
lys
CXWL01002797_10867 10865..273
ParE toxin
HTH-1
REP
ATP
MCP
lys
RHH
MDTD01008294_71312 11663..1
cell
cell
REP
ATP
MCP
S336
CEVF01306934_11223 11223..1
ATP
MCP
S336
B_PV_Planctomycetes_bacterium@OHB80712_MHYN01000336_12021 12021..1
ATP
MCP
S336
Glycosyl-hydrolase
New Rep protein
CXWK01061157_14355 1..14303
PolB
Glycosyltransferase
ATP
MCP
lys
LGOV01010410_12383 12125..170
PolB
S728
ATP
MCP
lys
contig_6000743_11054 2..10958
PolB
S728
ATP
MCP
lys
u_me_marine_sediment_metagenome@KKN30924_LAZR01002370_10985_10985 1..10697
S140
S400
ATP
MCP
B_PV_Phycisphaerae_bacterium@KPK75270_LJUC01000159_9151 1..9051
S140
S400
ATP
MCP
1000 nt

## Slide 5
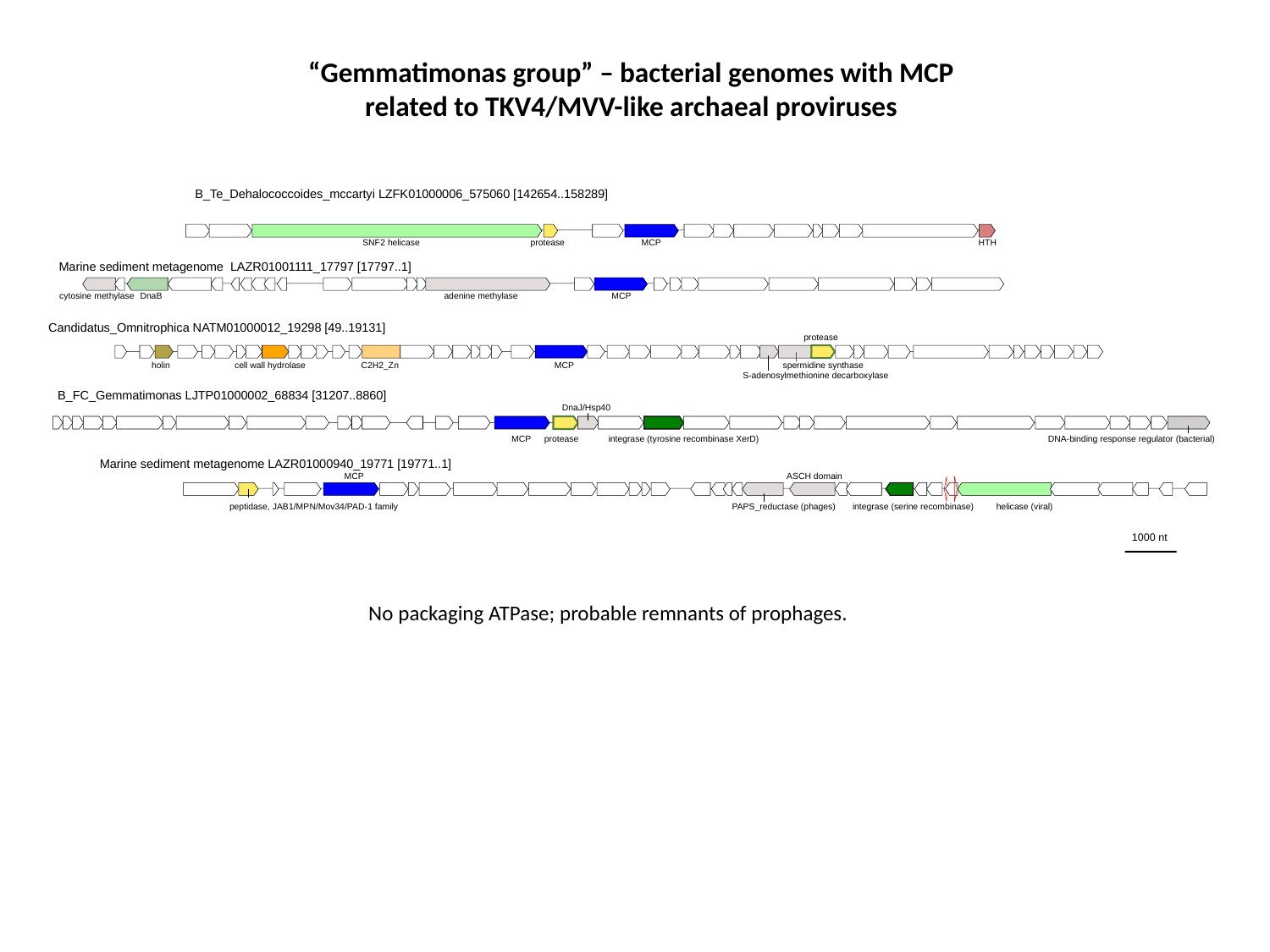

“Gemmatimonas group” – bacterial genomes with MCP related to TKV4/MVV-like archaeal proviruses
B_Te_Dehalococcoides_mccartyi LZFK01000006_575060 [142654..158289]
SNF2 helicase
protease
MCP
HTH
Marine sediment metagenome LAZR01001111_17797 [17797..1]
cytosine methylase
DnaB
adenine methylase
MCP
Candidatus_Omnitrophica NATM01000012_19298 [49..19131]
protease
spermidine synthase
holin
cell wall hydrolase
C2H2_Zn
MCP
S-adenosylmethionine decarboxylase
 B_FC_Gemmatimonas LJTP01000002_68834 [31207..8860]
DnaJ/Hsp40
MCP
protease
integrase (tyrosine recombinase XerD)
DNA-binding response regulator (bacterial)
Marine sediment metagenome LAZR01000940_19771 [19771..1]
MCP
ASCH domain
peptidase, JAB1/MPN/Mov34/PAD-1 family
PAPS_reductase (phages)
integrase (serine recombinase)
helicase (viral)
1000 nt
No packaging ATPase; probable remnants of prophages.
