# Supplementary material for: Vast diversity of prokaryotic virus genomes encoding double jelly-roll major capsid proteins uncovered by genomic and metagenomic sequence analysis
Source: Virol J. 2018 Apr 10;15:67. doi: 10.1186/s12985-018-0974-y (PMC5894146; doi:10.1186/s12985-018-0974-y)
Supplement: Supplementary file 7 — Bam35-Toil group genome maps. (PPTX 193 kb) [file 12985_2018_974_MOESM7_ESM.pptx]

## Slide 1
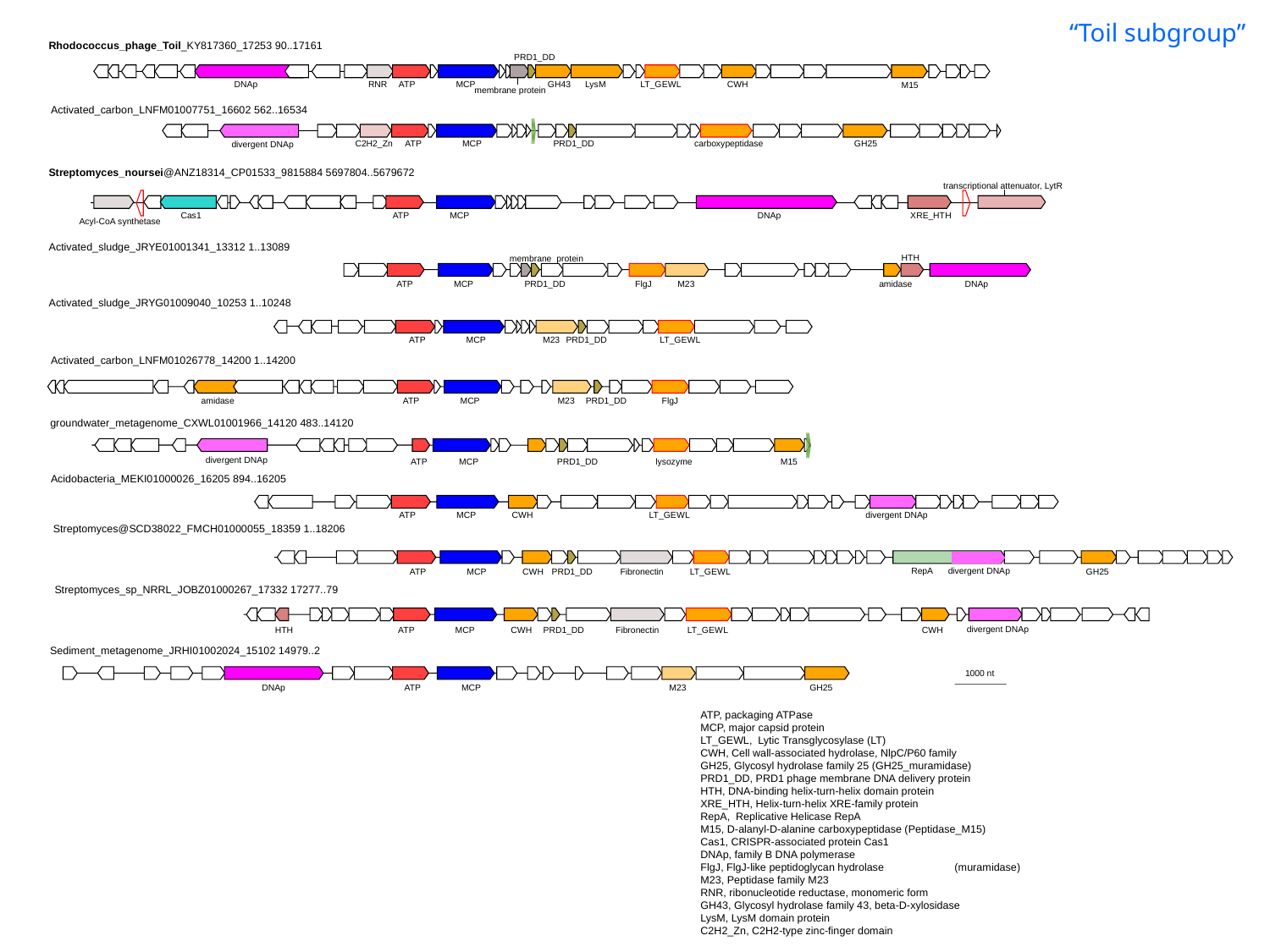

“Toil subgroup”
Rhodococcus_phage_Toil_KY817360_17253 90..17161
PRD1_DD
DNAp
RNR
ATP
MCP
GH43
LysM
LT_GEWL
CWH
membrane protein
M15
Activated_carbon_LNFM01007751_16602 562..16534
C2H2_Zn
ATP
MCP
PRD1_DD
carboxypeptidase
GH25
Streptomyces_noursei@ANZ18314_CP01533_9815884 5697804..5679672
transcriptional attenuator, LytR
Cas1
ATP
MCP
DNAp
XRE_HTH
Acyl-CoA synthetase
Activated_sludge_JRYE01001341_13312 1..13089
HTH
membrane protein
ATP
MCP
PRD1_DD
FlgJ
M23
amidase
DNAp
Activated_sludge_JRYG01009040_10253 1..10248
ATP
MCP
M23
PRD1_DD
LT_GEWL
Activated_carbon_LNFM01026778_14200 1..14200
amidase
ATP
MCP
M23
PRD1_DD
FlgJ
groundwater_metagenome_CXWL01001966_14120 483..14120
ATP
MCP
PRD1_DD
lysozyme
M15
Acidobacteria_MEKI01000026_16205 894..16205
ATP
MCP
CWH
LT_GEWL
Streptomyces@SCD38022_FMCH01000055_18359 1..18206
RepA
ATP
MCP
CWH
LT_GEWL
PRD1_DD
Fibronectin
GH25
Streptomyces_sp_NRRL_JOBZ01000267_17332 17277..79
HTH
ATP
MCP
CWH
LT_GEWL
PRD1_DD
Fibronectin
CWH
Sediment_metagenome_JRHI01002024_15102 14979..2
DNAp
ATP
MCP
M23
GH25
1000 nt
divergent DNAp
divergent DNAp
divergent DNAp
divergent DNAp
divergent DNAp
ATP, packaging ATPase
MCP, major capsid protein
LT_GEWL, Lytic Transglycosylase (LT)
CWH, Cell wall-associated hydrolase, NlpC/P60 family
GH25, Glycosyl hydrolase family 25 (GH25_muramidase)
PRD1_DD, PRD1 phage membrane DNA delivery protein
HTH, DNA-binding helix-turn-helix domain protein
XRE_HTH, Helix-turn-helix XRE-family protein
RepA, Replicative Helicase RepA
M15, D-alanyl-D-alanine carboxypeptidase (Peptidase_M15)
Cas1, CRISPR-associated protein Cas1
DNAp, family B DNA polymerase
FlgJ, FlgJ-like peptidoglycan hydrolase 	(muramidase)
M23, Peptidase family M23
RNR, ribonucleotide reductase, monomeric form
GH43, Glycosyl hydrolase family 43, beta-D-xylosidase
LysM, LysM domain protein
C2H2_Zn, C2H2-type zinc-finger domain

## Slide 2
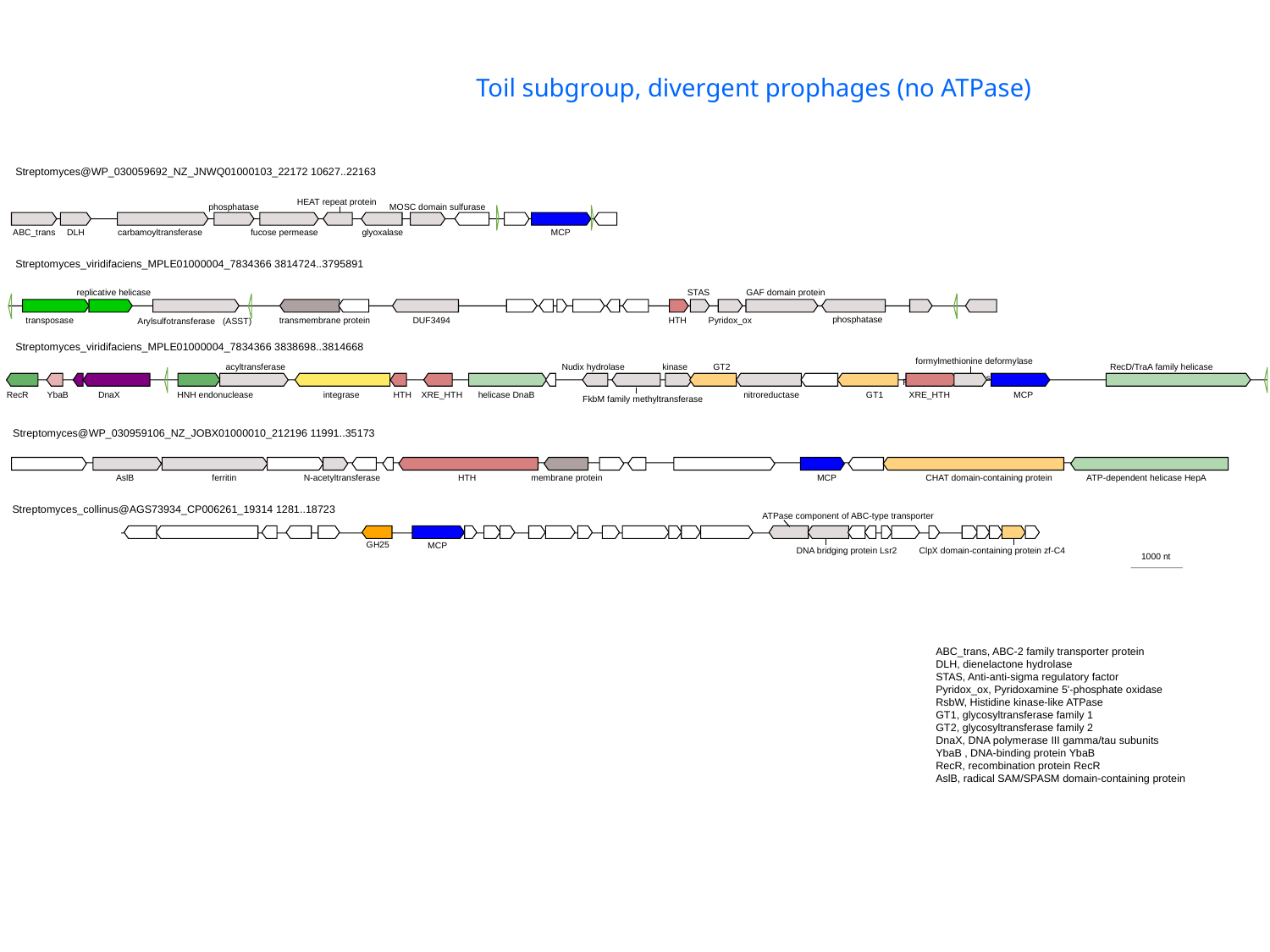

Toil subgroup, divergent prophages (no ATPase)
Streptomyces@WP_030059692_NZ_JNWQ01000103_22172 10627..22163
HEAT repeat protein
phosphatase
MOSC domain sulfurase
MCP
ABC_trans
DLH
carbamoyltransferase
fucose permease
glyoxalase
Streptomyces_viridifaciens_MPLE01000004_7834366 3814724..3795891
Arylsulfotransferase (ASST)
replicative helicase
STAS
GAF domain protein
phosphatase
transposase
transmembrane protein
DUF3494
HTH
Pyridox_ox
Streptomyces_viridifaciens_MPLE01000004_7834366 3838698..3814668
formylmethionine deformylase
acyltransferase
Nudix hydrolase
RecD/TraA family helicase
kinase
GT2
helicase DnaB
DnaX
FkbM family methyltransferase
RecR
YbaB
HNH endonuclease
integrase
HTH
XRE_HTH
nitroreductase
GT1
XRE_HTH
MCP
RsbW
cation transporter
Streptomyces@WP_030959106_NZ_JOBX01000010_212196 11991..35173
HTH
membrane protein
MCP
CHAT domain-containing protein
ATP-dependent helicase HepA
AslB
ferritin
N-acetyltransferase
Streptomyces_collinus@AGS73934_CP006261_19314 1281..18723
ATPase component of ABC-type transporter
GH25
MCP
DNA bridging protein Lsr2
ClpX domain-containing protein zf-C4
1000 nt
ABC_trans, ABC-2 family transporter protein
DLH, dienelactone hydrolase
STAS, Anti-anti-sigma regulatory factor
Pyridox_ox, Pyridoxamine 5'-phosphate oxidase
RsbW, Histidine kinase-like ATPase
GT1, glycosyltransferase family 1
GT2, glycosyltransferase family 2
DnaX, DNA polymerase III gamma/tau subunits
YbaB , DNA-binding protein YbaB
RecR, recombination protein RecR
AslB, radical SAM/SPASM domain-containing protein

## Slide 3
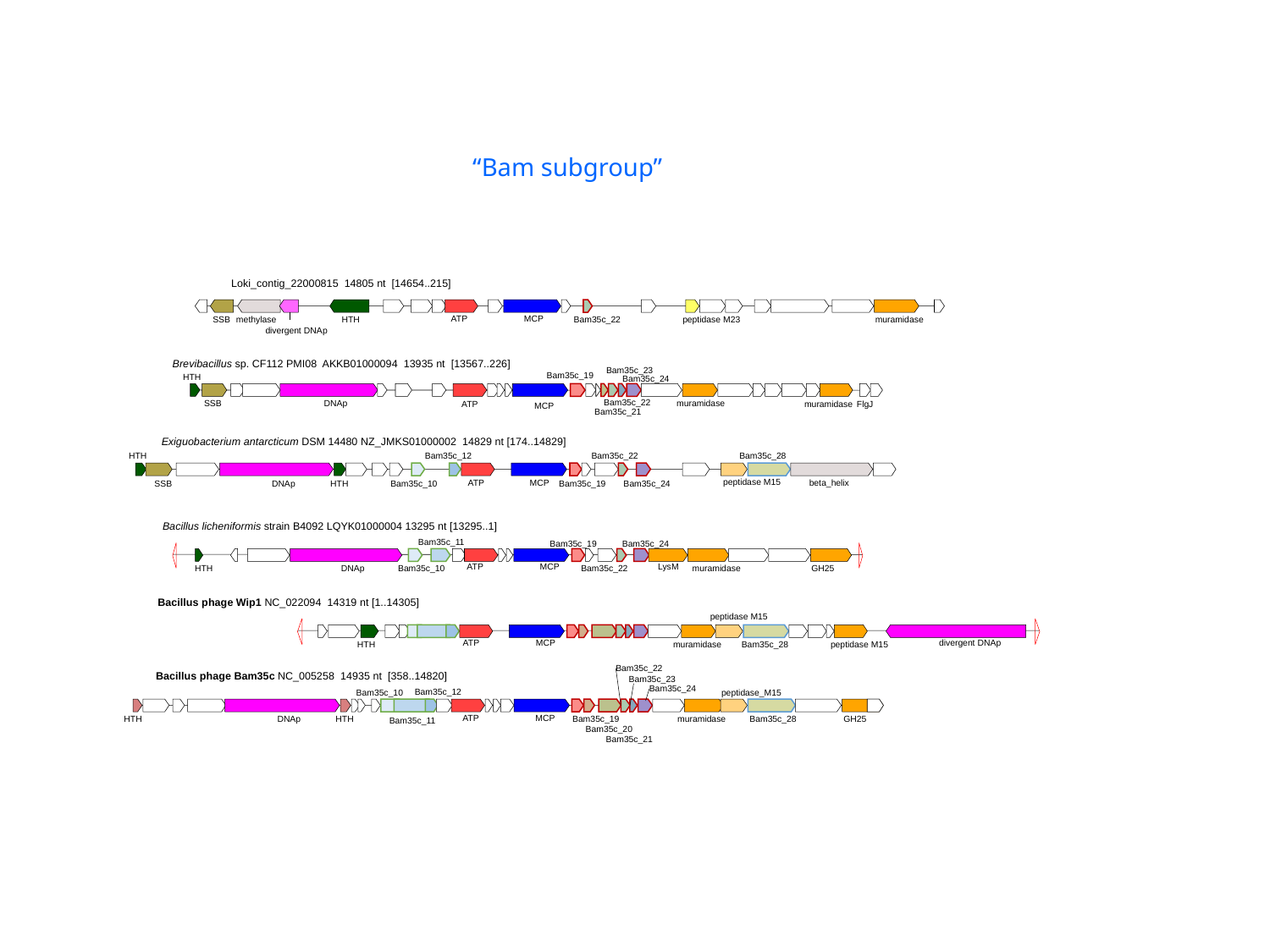

“Bam subgroup”
Loki_contig_22000815 14805 nt [14654..215]
ATP
MCP
SSB
methylase
HTH
Bam35c_22
peptidase M23
muramidase
divergent DNAp
Brevibacillus sp. CF112 PMI08 AKKB01000094 13935 nt [13567..226]
Bam35c_23
Bam35c_19
HTH
Bam35c_24
muramidase FlgJ
Bam35c_22
SSB
DNAp
muramidase
ATP
MCP
Bam35c_21
Exiguobacterium antarcticum DSM 14480 NZ_JMKS01000002 14829 nt [174..14829]
HTH
Bam35c_12
Bam35c_22
Bam35c_28
peptidase M15
ATP
MCP
beta_helix
SSB
DNAp
HTH
Bam35c_10
Bam35c_19
Bam35c_24
Bacillus licheniformis strain B4092 LQYK01000004 13295 nt [13295..1]
Bam35c_11
Bam35c_19
Bam35c_24
ATP
MCP
LysM
HTH
DNAp
Bam35c_10
Bam35c_22
muramidase
GH25
Bacillus phage Wip1 NC_022094 14319 nt [1..14305]
peptidase M15
ATP
MCP
divergent DNAp
HTH
muramidase
Bam35c_28
peptidase M15
Bam35c_22
Bam35c_23
Bam35c_24
Bam35c_12
peptidase_M15
Bam35c_10
ATP
MCP
Bam35c_19
HTH
DNAp
HTH
muramidase
Bam35c_28
GH25
Bam35c_11
Bam35c_20
Bam35c_21
Bacillus phage Bam35c NC_005258 14935 nt [358..14820]

## Slide 4
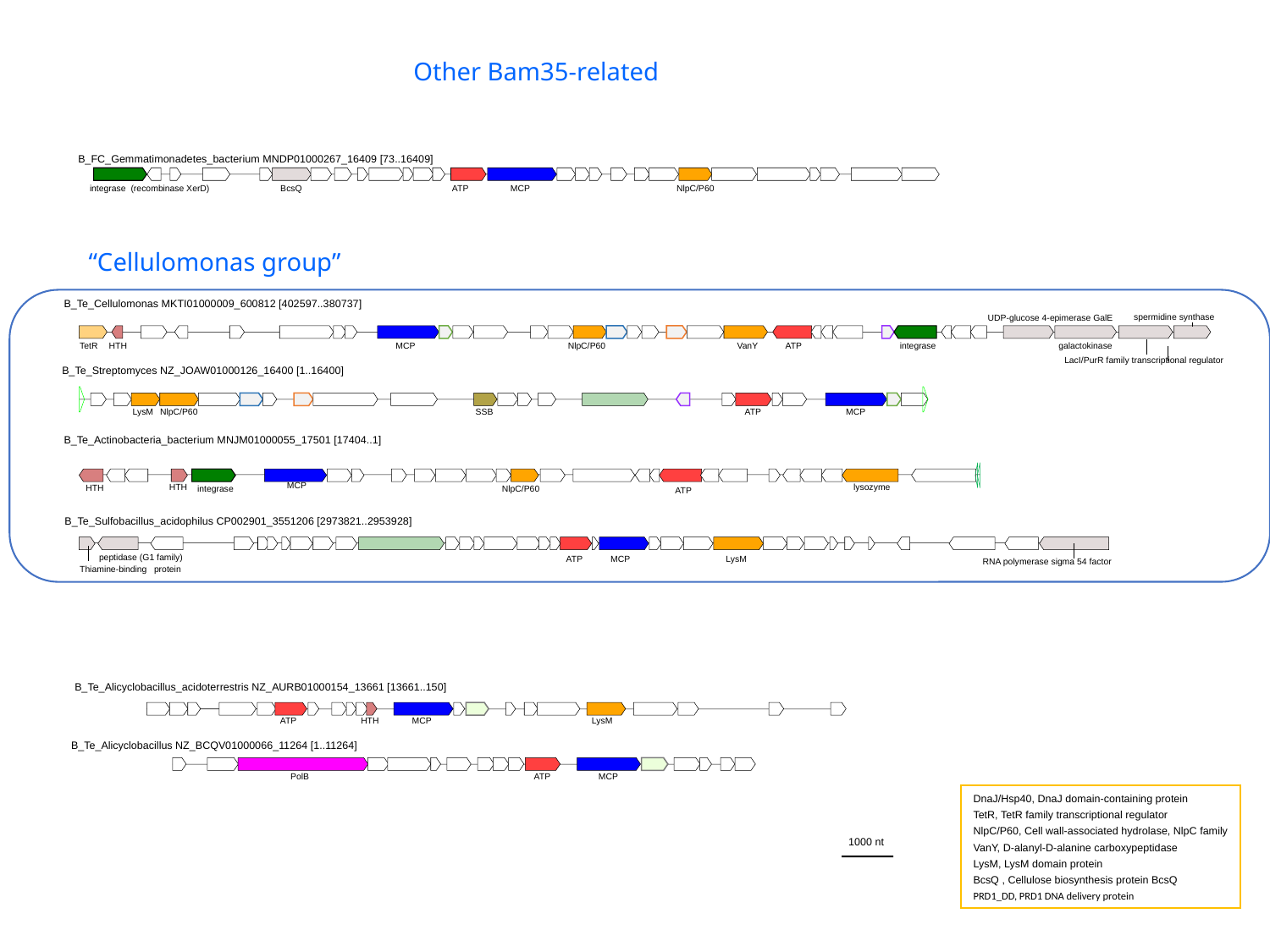

Other Bam35-related
B_FC_Gemmatimonadetes_bacterium MNDP01000267_16409 [73..16409]
integrase (recombinase XerD)
BcsQ
ATP
MCP
NlpC/P60
“Cellulomonas group”
B_Te_Cellulomonas MKTI01000009_600812 [402597..380737]
spermidine synthase
UDP-glucose 4-epimerase GalE
TetR
HTH
MCP
NlpC/P60
VanY
ATP
integrase
galactokinase
LacI/PurR family transcriptional regulator
B_Te_Streptomyces NZ_JOAW01000126_16400 [1..16400]
LysM
NlpC/P60
SSB
ATP
MCP
B_Te_Actinobacteria_bacterium MNJM01000055_17501 [17404..1]
MCP
lysozyme
HTH
HTH
NlpC/P60
integrase
ATP
B_Te_Sulfobacillus_acidophilus CP002901_3551206 [2973821..2953928]
Thiamine-binding protein
peptidase (G1 family)
ATP
MCP
LysM
RNA polymerase sigma 54 factor
B_Te_Alicyclobacillus_acidoterrestris NZ_AURB01000154_13661 [13661..150]
ATP
HTH
MCP
LysM
B_Te_Alicyclobacillus NZ_BCQV01000066_11264 [1..11264]
PolB
ATP
MCP
DnaJ/Hsp40, DnaJ domain-containing protein
TetR, TetR family transcriptional regulator
NlpC/P60, Cell wall-associated hydrolase, NlpC family
VanY, D-alanyl-D-alanine carboxypeptidase
LysM, LysM domain protein
BcsQ , Cellulose biosynthesis protein BcsQ
PRD1_DD, PRD1 DNA delivery protein
1000 nt
