# Supplementary material for: Vast diversity of prokaryotic virus genomes encoding double jelly-roll major capsid proteins uncovered by genomic and metagenomic sequence analysis
Source: Virol J. 2018 Apr 10;15:67. doi: 10.1186/s12985-018-0974-y (PMC5894146; doi:10.1186/s12985-018-0974-y)
Supplement: Supplementary file 8 — PRD1 group MCP tree. (PPTX 36 kb) [file 12985_2018_974_MOESM8_ESM.pptx]

## Slide 1
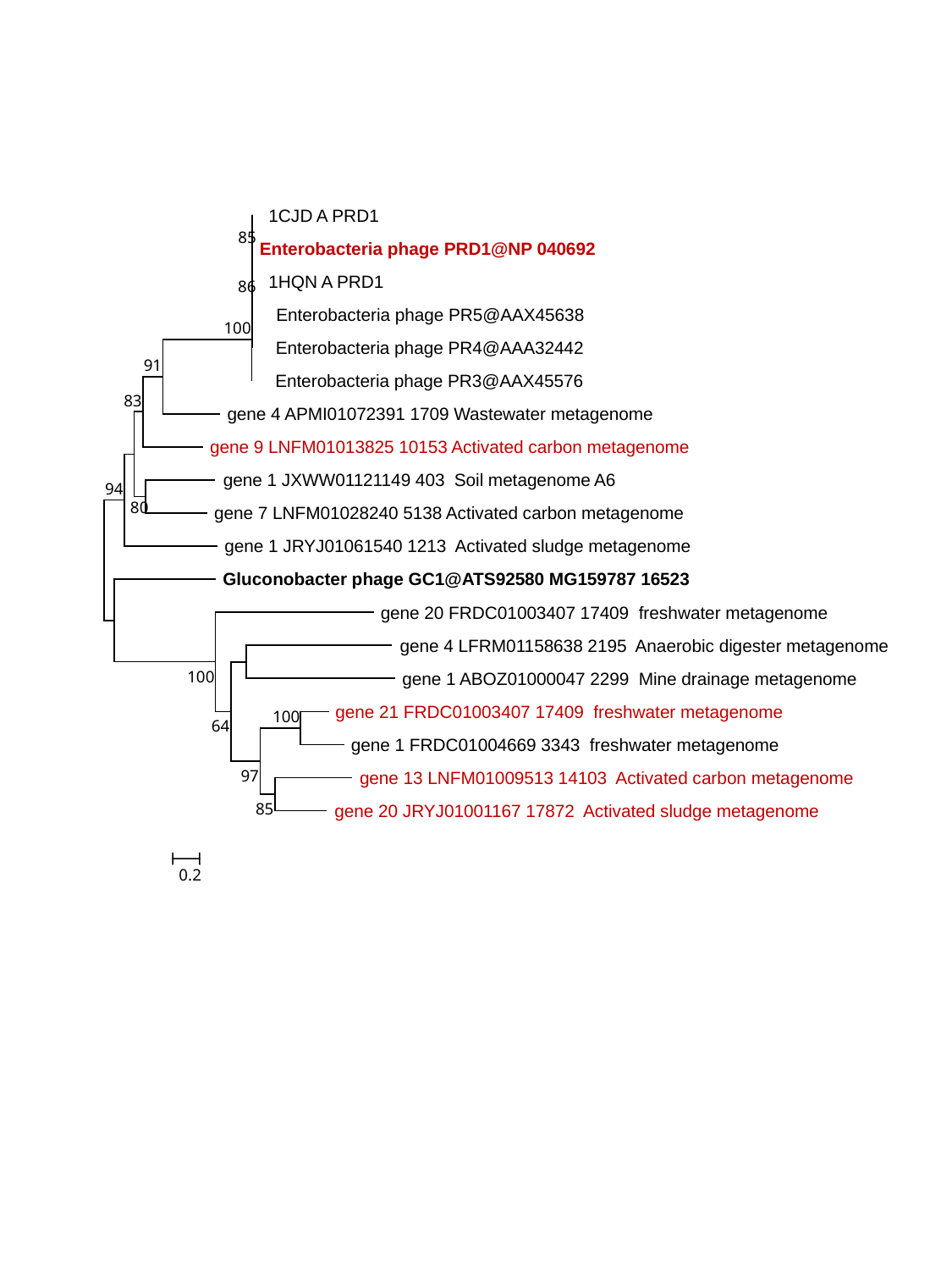

1CJD A PRD1
85
 Enterobacteria phage PRD1@NP 040692
 1HQN A PRD1
86
 Enterobacteria phage PR5@AAX45638
100
 Enterobacteria phage PR4@AAA32442
91
 Enterobacteria phage PR3@AAX45576
83
 gene 4 APMI01072391 1709 Wastewater metagenome
 gene 9 LNFM01013825 10153 Activated carbon metagenome
 gene 1 JXWW01121149 403 Soil metagenome A6
94
80
 gene 7 LNFM01028240 5138 Activated carbon metagenome
 gene 1 JRYJ01061540 1213 Activated sludge metagenome
 Gluconobacter phage GC1@ATS92580 MG159787 16523
 gene 20 FRDC01003407 17409 freshwater metagenome
 gene 4 LFRM01158638 2195 Anaerobic digester metagenome
100
 gene 1 ABOZ01000047 2299 Mine drainage metagenome
 gene 21 FRDC01003407 17409 freshwater metagenome
100
64
 gene 1 FRDC01004669 3343 freshwater metagenome
97
 gene 13 LNFM01009513 14103 Activated carbon metagenome
85
 gene 20 JRYJ01001167 17872 Activated sludge metagenome
0.2
